# Author Correction: Elevated plasma complement factor H related 5 protein is associated with venous thromboembolism

**DOI:** 10.1038/s41467-023-43764-4

**Published:** 2023-11-27

**Authors:** Maria Jesus Iglesias, Laura Sanchez-Rivera, Manal Ibrahim-Kosta, Clément Naudin, Gaëlle Munsch, Louisa Goumidi, Maria Farm, Philip M. Smith, Florian Thibord, Julia Barbara Kral-Pointner, Mun-Gwan Hong, Pierre Suchon, Marine Germain, Waltraud Schrottmaier, Philip Dusart, Anne Boland, David Kotol, Fredrik Edfors, Mine Koprulu, Maik Pietzner, Claudia Langenberg, Scott M. Damrauer, Andrew D. Johnson, Derek M. Klarin, Nicholas L. Smith, David M. Smadja, Margareta Holmström, Maria Magnusson, Angela Silveira, Mathias Uhlén, Thomas Renné, Angel Martinez-Perez, Joseph Emmerich, Jean-Francois Deleuze, Jovan Antovic, Jose Manuel Soria Fernandez, Alice Assinger, Jochen M. Schwenk, Joan Carles Souto Andres, Pierre-Emmanuel Morange, Lynn Marie Butler, David-Alexandre Trégouët, Jacob Odeberg

**Affiliations:** 1 Science for Life Laboratory, Department of Protein Science, CBH, KTH Royal Institute of Technology, SE-171 21, Stockholm, Sweden; 2 Division of Internal Medicine, University Hospital of North Norway (UNN), PB100, 9038 Tromsø, Norway; 3 Translational Vascular Research, Department of Clinical Medicine, UiT The Arctic University of Norway, 9019 Tromsø, Norway; 4 Aix-Marseille Univ, INSERM, INRAE, C2VN, Laboratory of Haematology, CRB Assistance Publique—Hôpitaux de Marseille, HemoVasc (CRB AP-HM HemoVasc), Marseille, France; 5 University of Bordeaux, INSERM, Bordeaux Population Health Research Center, UMR 1219 ELEANOR, Bordeaux, France; 6 Department of Molecular Medicine and Surgery, Karolinska Institute, Stockholm, Sweden; 7 Department of Clinical Chemistry, Karolinska University Hospital, Stockholm, Sweden; 8 Department of Medicine Solna, Karolinska Institute and Karolinska University Hospital, Stockholm, Sweden; 9 Theme of Emergency and Reparative Medicine, Karolinska University Hospital, Stockholm, Sweden; 10 Population Sciences Branch, Division of Intramural Research, National Heart, Lung and Blood Institute, Framingham, MA USA; 11 The Framingham Heart Study, Boston University, Framingham, MA USA; 12 Center for Physiology and Pharmacology, Institute of Vascular Biology and Thrombosis Research, Medical University of Vienna, Vienna, Austria; 13 Laboratory of Excellence GENMED (Medical Genomics), Bordeaux, France; 14 Université Paris-Saclay, CEA, Centre National de Recherche en Génomique Humaine (CNRGH), 91057 Evry, France; 15 Laboratory of Excellence GENMED (Medical Genomics), Evry, France; 16 MRC Epidemiology Unit, University of Cambridge School of Clinical Medicine, Institute of Metabolic Science, Cambridge, CB2 0QQ UK; 17 Computational Medicine, Berlin Institute of Health at Charité-Universitätsmedizin Berlin, 10117 Berlin, Germany; 18 Precision Healthcare University Research Institute, Queen Mary University of London, London, UK; 19 Corporal Michael Crescenz VA Medical Center, Philadelphia, PA USA; 20 Department of Surgery and Department of Genetics, Perelman School of Medicine, University of Pennsylvania, Philadelphia, PA USA; 21 VA Palo Alto Healthcare System, Palo Alto, CA USA; 22 Department of Vascular Surgery, Stanford University School of Medicine, Palo Alto, CA USA; 23 Department of Epidemiology, University of Washington, Seattle, WA USA; 24 Kaiser Permanente Washington Health Research Institute, Seattle, WA USA; 25 Seattle Epidemiologic Research and Information Center, Department of Veterans Affairs Office of Research and Development, Seattle, WA USA; 26 Hematology Department and Biosurgical Research Lab (Carpentier Foundation), European Georges Pompidou Hospital, Assistance Publique Hôpitaux de Paris, 20 rue Leblanc, Paris, 75015 France; 27 Innovative Therapies in Haemostasis, INSERM, Université de Paris, 4 avenue de l’Observatoire, Paris, 75270 France; 28 Coagulation Unit, Department of Haematology, Karolinska University Hospital, SE-171 76, Stockholm, Sweden; 29 Department of Clinical Science, Intervention and Technology, Karolinska Institute, 171 77, Stockholm, Sweden; 30 Institute for Clinical Chemistry and Laboratory Medicine, University Medical Centre Hamburg-Eppendorf, D-20246 Hamburg, Germany; 31 Center for Thrombosis and Hemostasis (CTH), Johannes Gutenberg University Medical Center, D-55131 Mainz, Germany; 32 Irish Centre for Vascular Biology, School of Pharmacy and Biomolecular Sciences, Royal College of Surgeons in Ireland, Dublin 2, D02 YN77 Ireland; 33 Genomics of Complex Diseases Group, Research Institute Hospital de la Santa Creu i Sant Pau. IIB Sant Pau, Barcelona, Spain; 34 Department of vascular medicine, Paris Saint-Joseph Hospital Group, INSERM 1153-CRESS, University of Paris Cité, 185 rue Raymond Losserand, Paris, 75674 France; 35 Centre D’Etude du Polymorphisme Humain, Fondation Jean Dausset, Paris, France; 36 Unitat d’Hemostàsia i Trombosi, Hospital de la Santa Creu i Sant Pau and IIB-Sant Pau, Barcelona, Spain

**Keywords:** Thromboembolism, Diagnostic markers, Predictive markers, Risk factors

Correction to: *Nature Communications* 10.1038/s41467-023-38383-y, published online 07 June 2023

In this article the author name Waltraud Schrottmaier was incorrectly written as Waltraud Schottmaier. The original article has been corrected.

