# Supplementary material for: Elevated plasma complement factor H related 5 protein is associated with venous thromboembolism
Source: Nat Commun. 2023 Jun 7;14:3280. doi: 10.1038/s41467-023-38383-y (PMC10247781; doi:10.1038/s41467-023-38383-y)

Figure S1

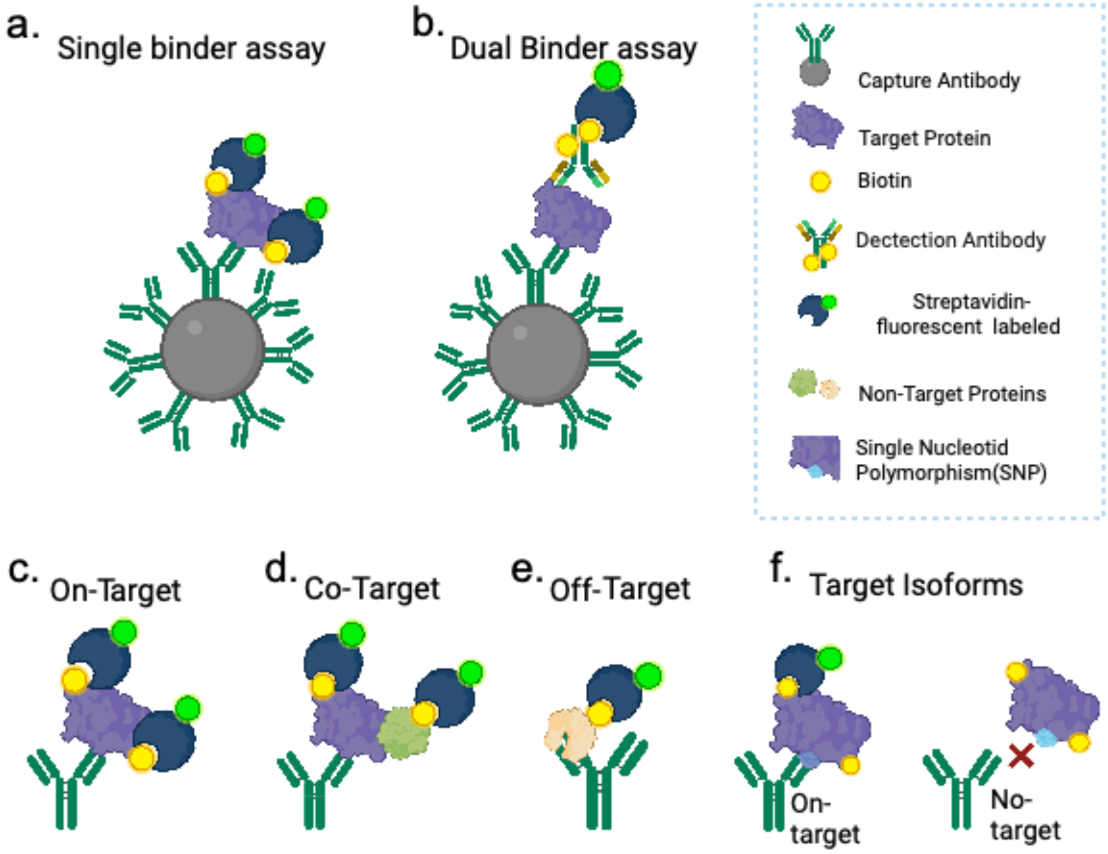

Figure S1: Antibody based suspension bead assay concepts. The antibody raised towards intended protein target is covalently coupled to colour-coded, micrometer-sized beads. (**a**) In the single binder assay, all proteins within the plasma sample are labelled with biotin before incubation with beads. Biotin-labelled proteins bound to the capture antibody are detected by fluorescent-labelled-streptavidin. The suspension is analysed by a cytometry-based instrument (Luminex), where the colour of the antibody-coupled bead provides the antibody ID, and the mean fluorescence intensity provides a relative measure of the bound target protein corresponding to plasma levels. (**b**) In the dual binder assay captured proteins are unlabelled, and detection of target protein bound to the capture antibody is through a secondary target-specific biotin-labelled detection antibody, followed by fluorescent-labelled-streptavidin addition and analysis on a Luminex instrument. As detection in the single binder assay is based on detection of biotin bound directly to target proteins, the signal can reflect either (**c**) the intended on-target binding, (**d**) co-target binding where the target protein is complexed with non-target protein or (**e**) off-target binding, i.e., binding of a non-target protein. (**f**) An amino-acid substitution in the epitope of the target protein can lead to isoform specific binding.

Figure S2

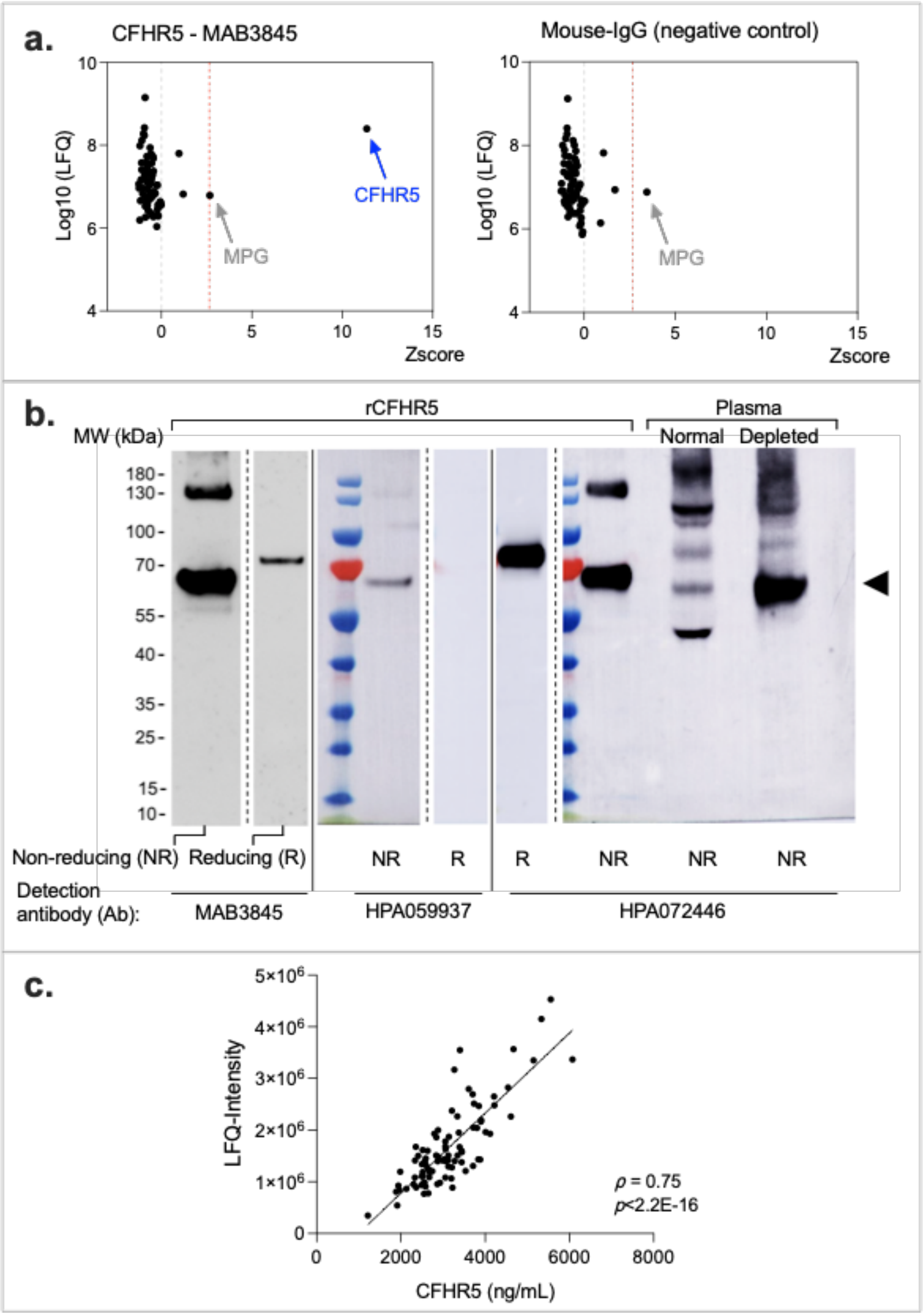
a.
CFHR5 - MAB3845
Mouse-IgG (negative control)
Log10 (LFQ)
Zscore
CFHR5
MPG
b.
MW (kDa)
rCFHR5
Plasma
Normal
Depleted
180-
130-
100-
70-
55-
40-
35-
25-
15-
10-
Non-reducing (NR)
Reducing (R)
NR
R
R
NR
NR
NR
Detection antibody (Ab):
MAB3845
HPA059937
HPA072446
c.
LFQ-Intensity
CFHR5 (ng/mL)
ρ = 0.75
p<2.2E-16

Figure S2: Verification of antibody target and CFHR5 dual binder quantitative assay **(a)** Immunocapture mass spectrometry analysis of monoclonal anti-CFHR5 detection antibody (MAB3845, clone#390513; 1,76 µg per incubation) and negative control (Mouse IgG,1,76 µg) (*n*=3 of independent biological replicates). **(b)** Immunodetection of CFHR5 by Western blot (*n*=2). Recombinant CFHR5 (rCFHR5, 100 ng), pooled normal plasma (from 59 donors: 31 males, 28 females) (Normal, 1 µl) and 14 most abundant proteins depleted pooled plasma (from 59 individuals, 31 males, 28 females) (Depleted, 10 µl) loaded on SDS PAGE in non-reducing (without DTT, **NR**) or reducing conditions (with DTT, **R**). After electrophoresis and transfer on PVDF membrane, protein was detected using antibodies against SULF1 (HPA059937) and CFHR5 (HPA072446 and MAB3845). The arrow shows the band corresponding to CFHR5 protein. **(c)** Two-sided Spearman´s correlation analysis between quantitative dual binding assay and label-free quantitative data-independent acquisition mass spectrometry data (LFQ-DIA-MS) in VEBIOS ER (*n*=96; *p*<2.2E-16). Source data are provided as a Source Data file (tabs:FigS2a,FigS2b&FigS2c). LFQ: Label Free Quantification, **NR**: Non reducing conditions, **R**: Reducing conditions.

Figure S3

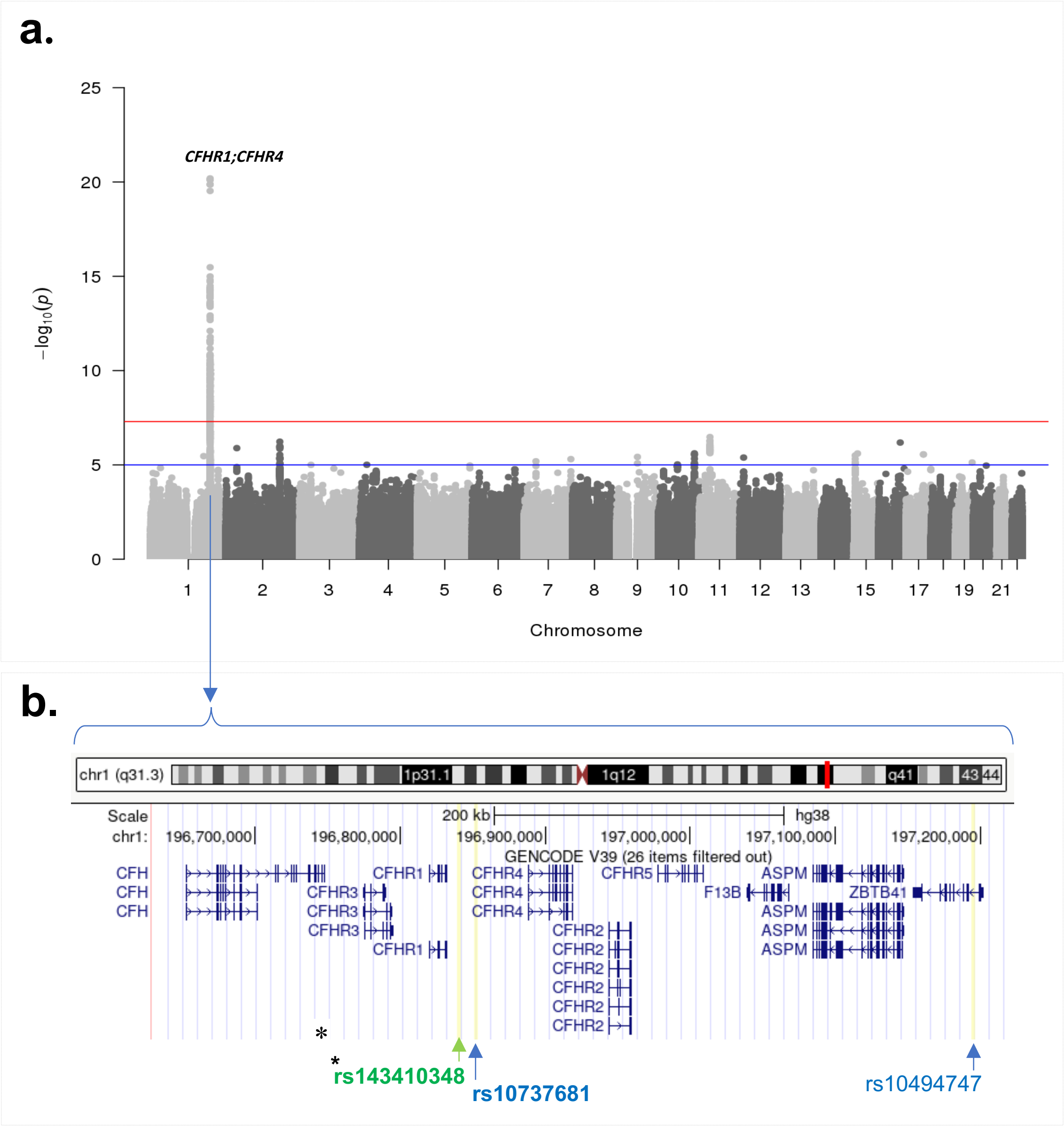

Figure S3: GWAS analysis identifies a CFHR5 pQTL on Chromosome 1 q31.3. A meta-analysis of GWAS data for 7,135,343 SNPs tested for association with CFHR5 concentrations in a total sample of 2967 individuals from the FARIVE, MARTHA and RETROVE studies: **(a)** identified one genome-wide significant (p<5E-08) signal on chr1q31.3. **(b)** The lead SNP at this locus, rs10737681, maps between the *CFHR1* and *CFHR4* gene loci in the gene cluster of *CFHR1-5*. A borderline significant association (p=9.83E-08) with CFHR5 levels was observed at the rs10494747, mapping to the *ZBTB41* gene. *Indicates (rs143410348) recently identified with genome wide significance as associated with VTE risk [17].

Figure S4

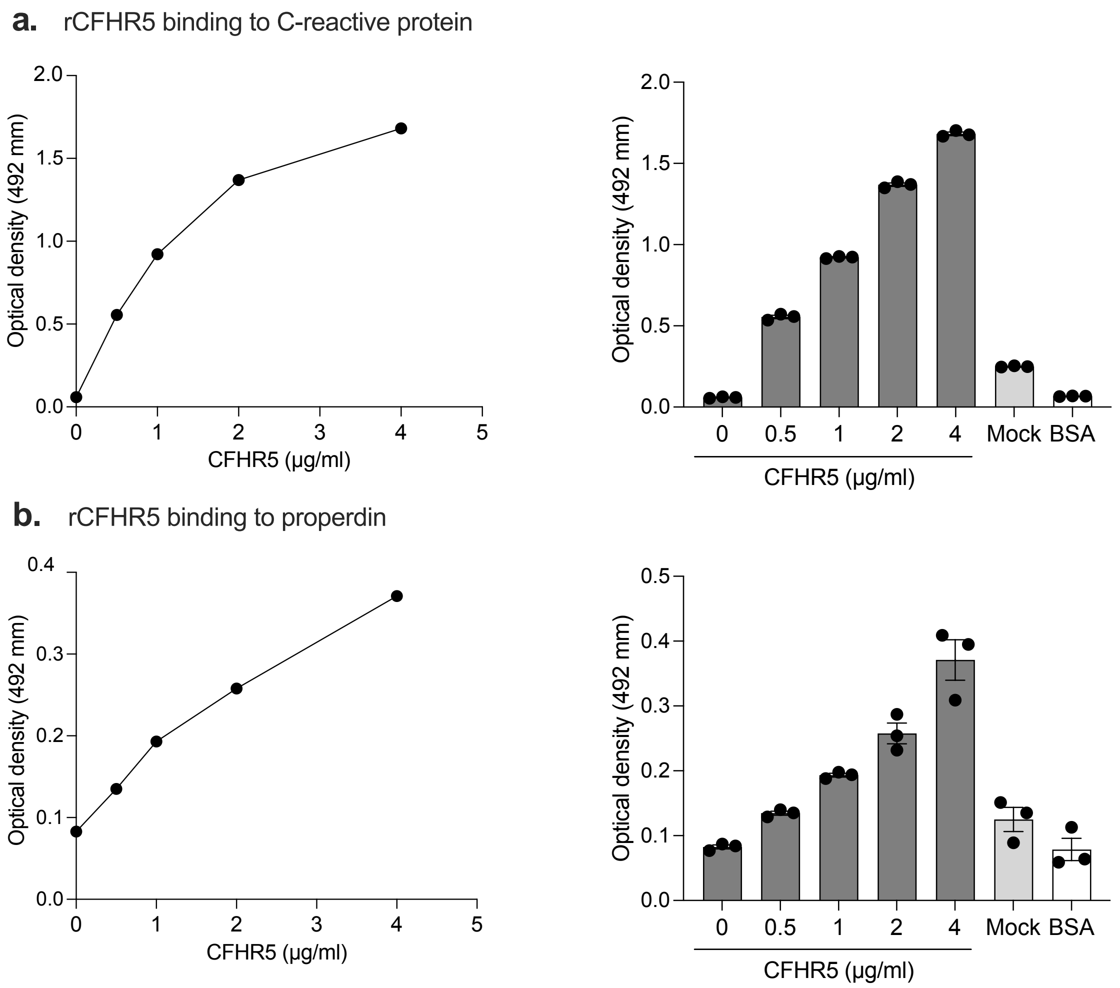

Figure S4. Recombinant CFHR5 (rCFHR5) binding to C-reactive protein (CRP) and Properdin. (**a**) CRP or (**b**) properdin, were immobilized on microtiter plates, before serial dilutions of rCFHR5 were added and binding assayed by ELISA. Data is displayed as mean signal across serial dilution (left panel) or measurements for individual replicates and controls (right panel) (mock; heat-denatured rCFHR5, BSA; bovine serum albumin). (*n*=3 for both experimental conditions). Histograms are represented as mean with standard error of the mean (SEM). Source data are provided as a Source Data file (tab:FigS4).

Figure S5

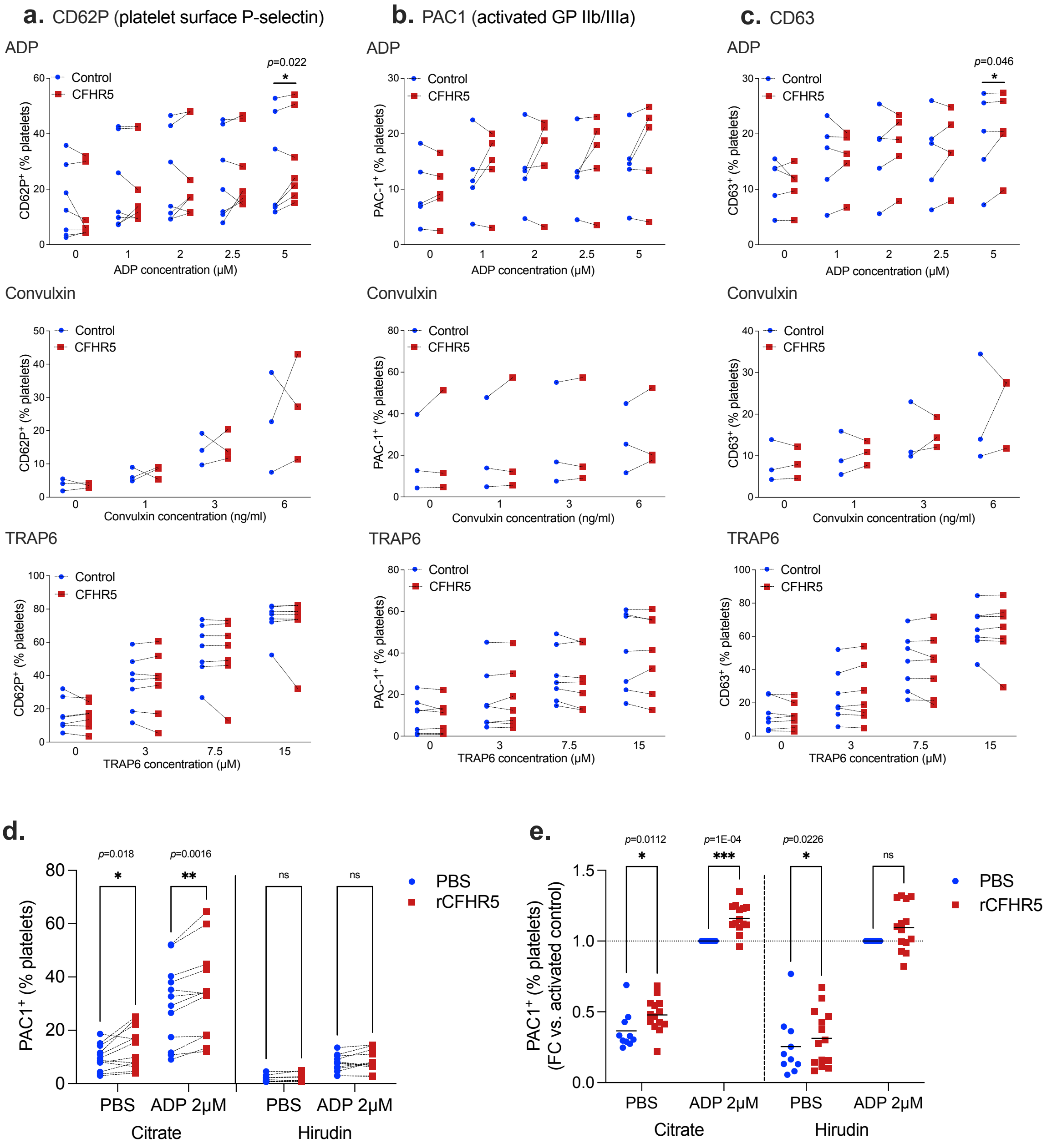

Figure S5: Recombinant CFHR5 does not potentiate platelet activation of washed platelets. Platelet activation was measured by surface expression of (**a**) P-selectin (left), (**b**) activated GP IIb/IIIa (PAC1$^+$)(middle) or (**c**) CD63 (right), following treatment of washed platelets with different concentrations of: adenosine diphosphate (ADP)(top), convulxin (midde) or TRAP6 (bottom), following preincubation (10 minutes) with 6 μg/ml recombinant CFHR5, or PBS control. (**d**) Baseline or ADP-induced platelet activated GP IIb/IIIa (PAC1+) expression (%) was measured on platelets in plasma isolated from citrate- or hirudin-anticoagulated blood, following preincubation with either PBS control or recombinant CFHR5. The same data, normalized to each respective ADP-stimulated control, is presented in (**e**). US: unstimulated, control (PBS). Each experiment is represented by an individual point and paired experiments connected by a dotted line. (5a-e) ANOVA two-sided test were performed. *p<0.05 **p<0.01 ***p<0.001. Source data are provided as a Source Data file (tab:FigS5 a-e)

Figure S6

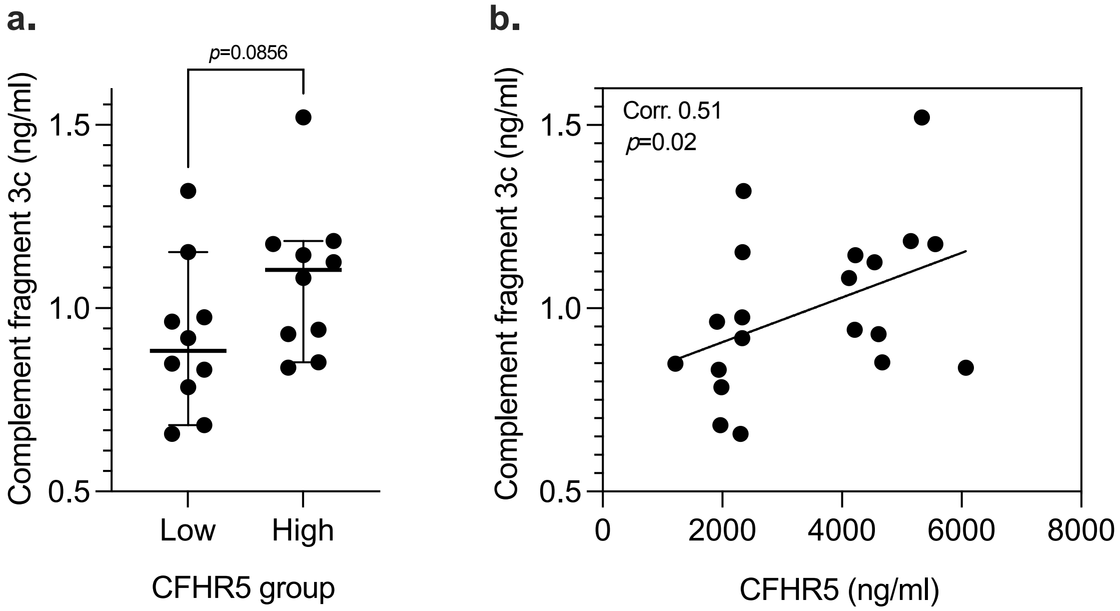

Figure S6. Relationship between CFHR5 and C3c levels in *VEBIOS ER* sample sub-set
Concentration of complement fragment 3c (C3c) was measured by ELISA in 20 VEBIOS ER cohort samples, which were selected based on high or low plasma concentration of CFHR5 (low CFHR5 group [<2500 ng/ml, *n*=10, female=6, male=4]; high CFHR5 group [>3800 ng/ml, *n*=10, female=5, male=5]). Data is presented by **(a)** CFHR5 group and **(b)** correlation between CFHR5 and C3c across all analysed samples. Dot plot data is presented as median with 95% CI (confidence interval). (6a) *t* test, two-sided, *p* value. (6b). Corr.: spearman´s correlation. Source data are provided as a Source Data file (tab:FigS6).

Figure S7

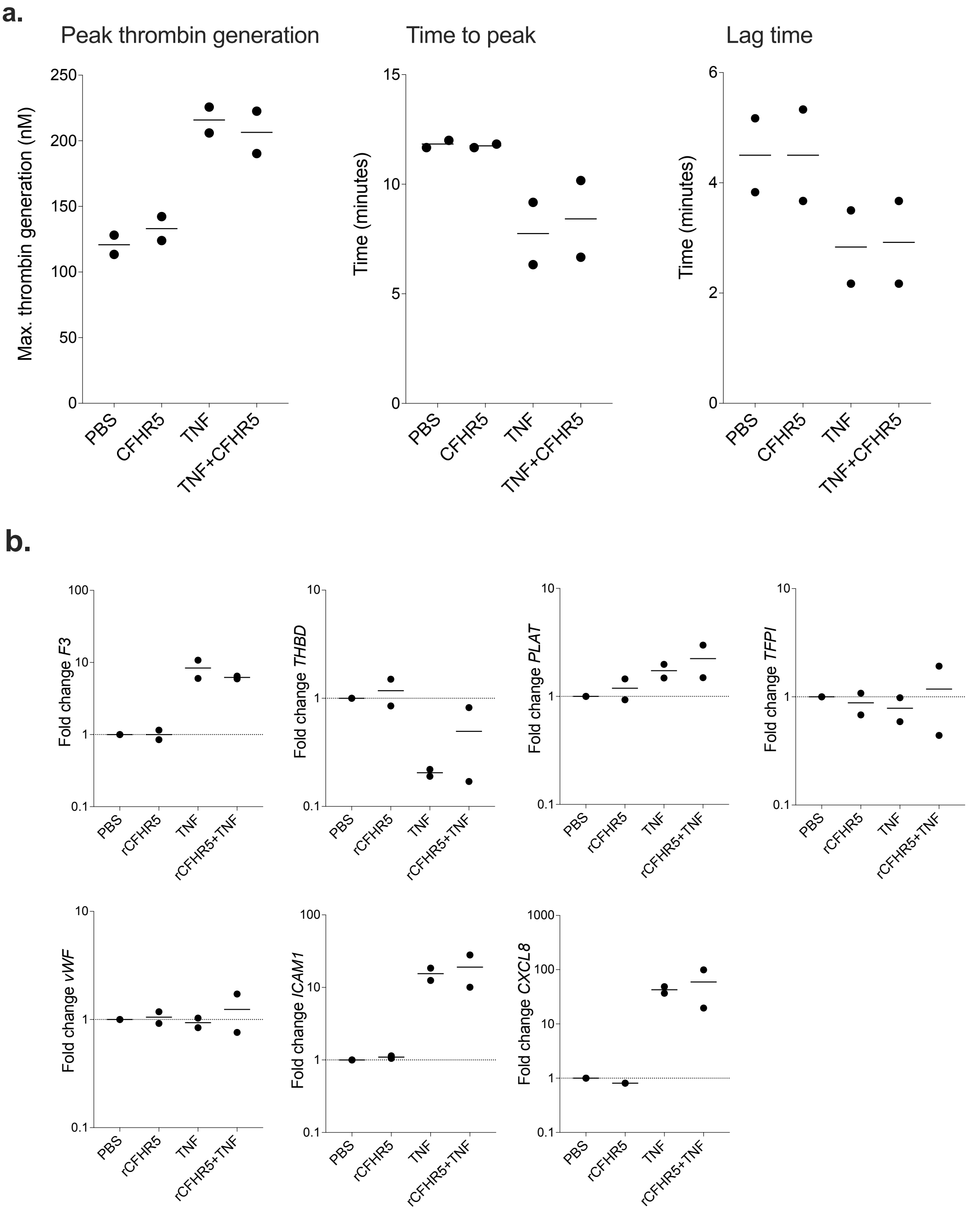

Figure S7: Effect of CFHR5 on HUVEC coagulability and inflammatory response. HUVEC were stimulated with rCFHR5 (6 µg/ml), the inflammatory cytokine tumour necrosis factor alpha (TNF, Sigma) (10 ng/ml), both or neither (PBS controls), for 24h before measurement of: **(a)** thrombin formation, assessed by real-time thrombin formation assay (*n*=2) and parameters of thrombin generation (Peak, Time to peak and Lag time) determined by the thrombinoscope software, or **(b)** relative gene expression of *F3, THBD, PLAT, TFPI, vWF, ICAM1 and CXCL8*, measured by real-time quantitative PCR (*n*=2). Data presented individual independent experiments with mean values**.** Source data are provided as a Source Data file (tab: Fig S7). F3: Tissue factor, *THBD*: thrombomodulin, *PLAT*: plasminogen activator, tissue type, *TFPI*: tissue factor pathway inhibitor, *vWF:* von Willebrand Factor, *ICAM1*: intracellular adhesion molecule 1, *CXCL8*: interleukin 8.

Supplementary information

Figure S8

Top panel

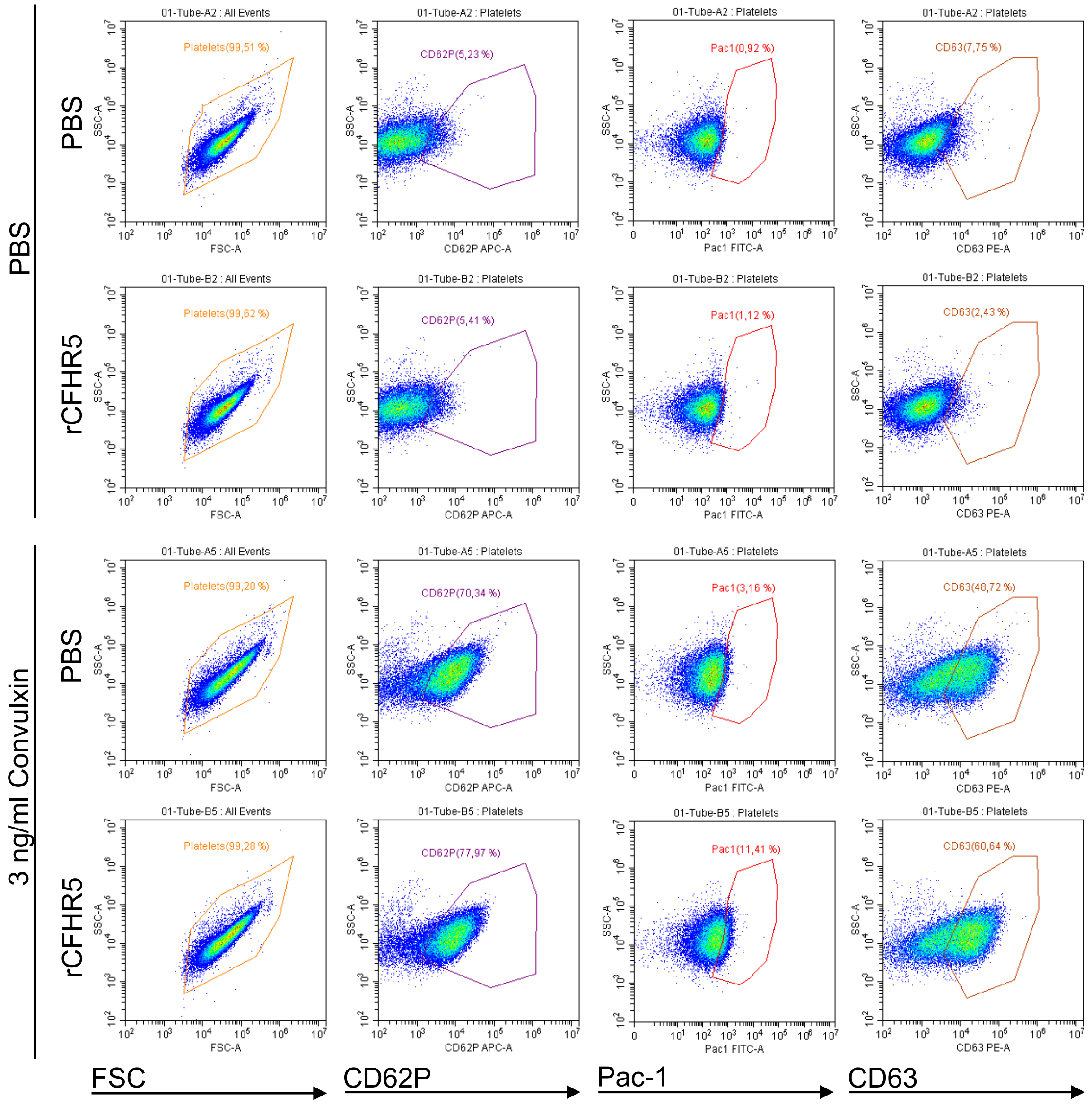

Bottom panel

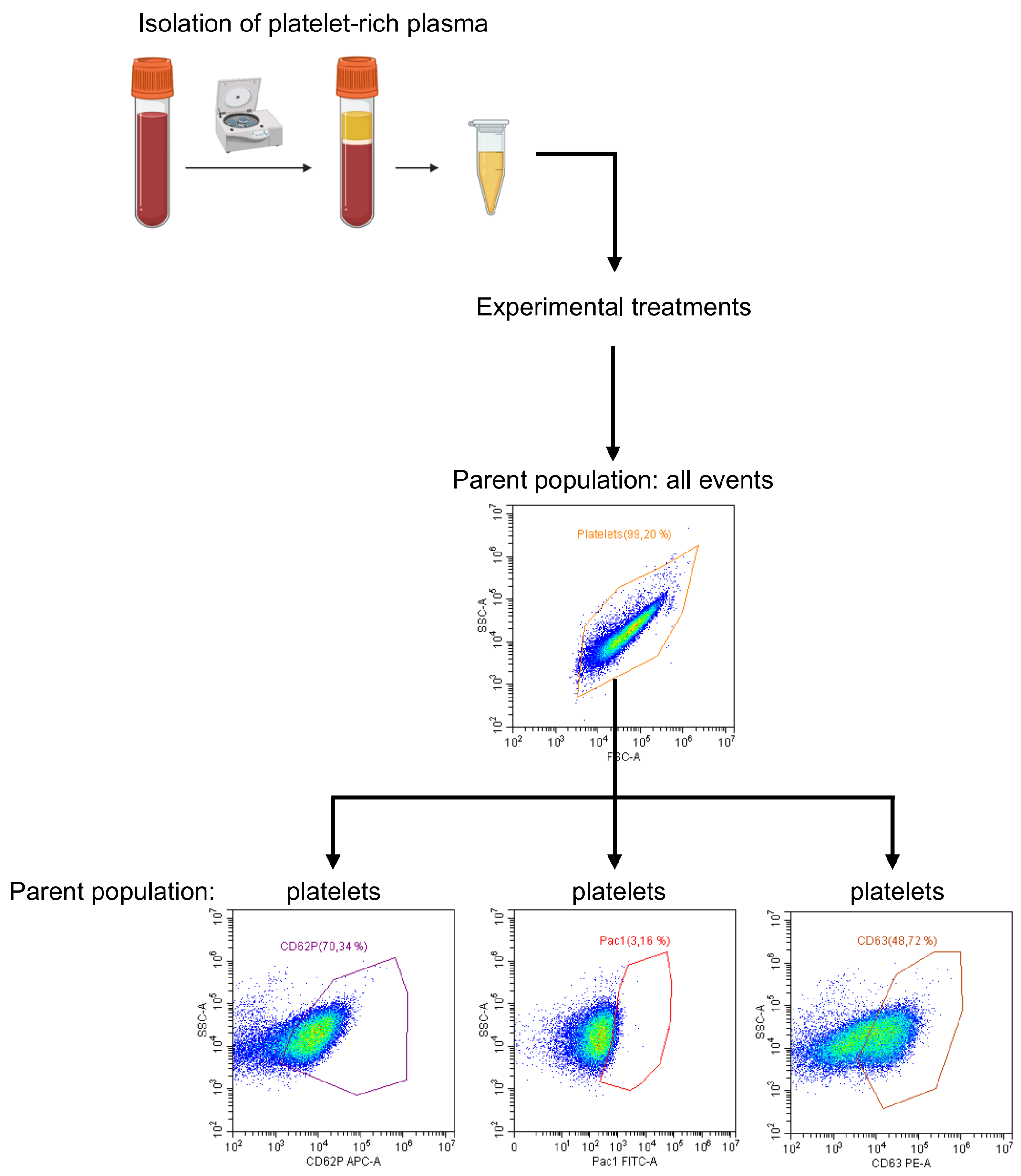

Figure S8: Gating strategy for the analysis of platelet activation of Figure 5 and S5. Top panel: Frist, platelets were identified according to their forward (FSC) and side scatter (SSC, x-axis of all blots). More than 99% of the cell population were platelets. Subsequently, expression of different platelet activation markers (CD62P, Pac-1, CD63) were determined. Representative flow cytometer blots are given under basal (PBS) and activated (3 ng/ml convulxin) conditions with or without rCFHR5. **Bottom panel:** Platelet-rich plasma was obtained from whole blood by centrifugation, thereby removing the vast majority of leukocytes and erythrocytes. Upon flow cytometric analysis platelets were identified according to their forward (FSC) and side scatter (SSC, x-axis of all blots). More than 99% of the cell population were platelets. Subsequently, expression of different platelet activation markers (CD62P, Pac-1, CD63) were determined.

Supplementary information for S2

Original compiled panel (Figure S2)

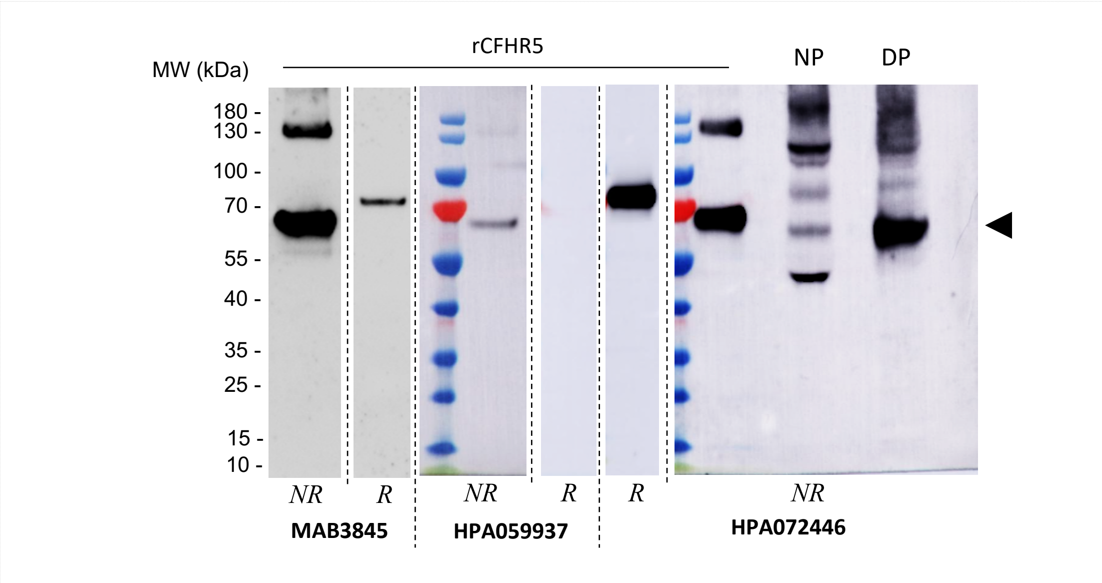

Uncropped gels:

slice 1 and 2

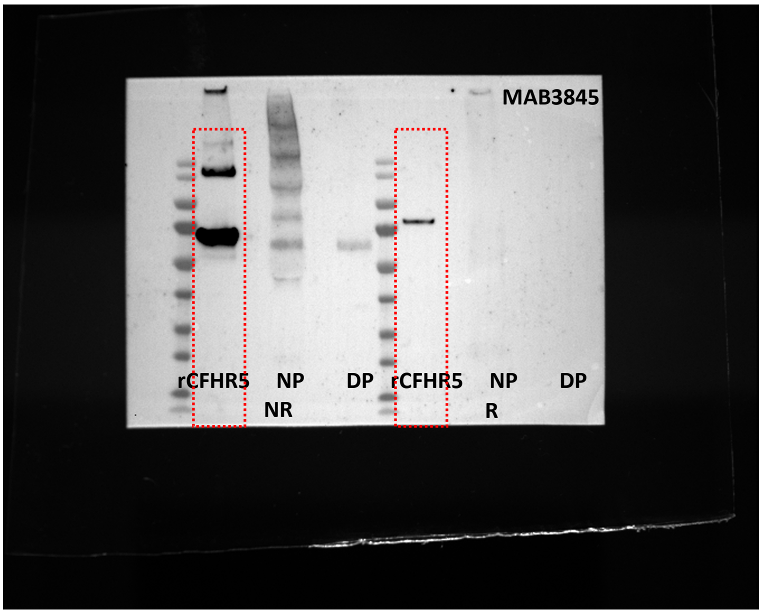

NP: Normal plasma; DP: Depleted plasma
NR: Non-reducing condition; R: Reducing condition

Slice 3: HPA059937 and Slice 6: HPA072446

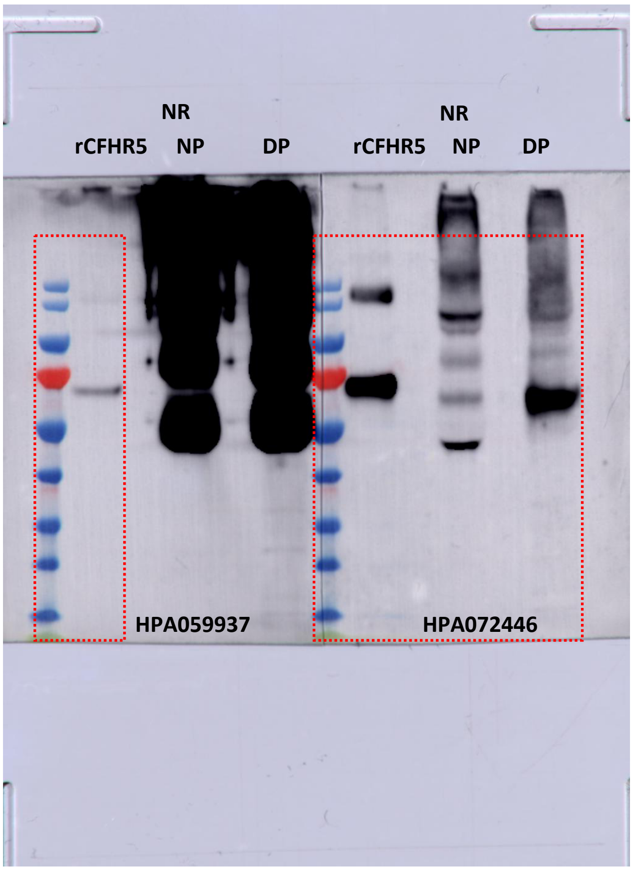

Slice 4: HPA059937 and Slice 5: HPA072446

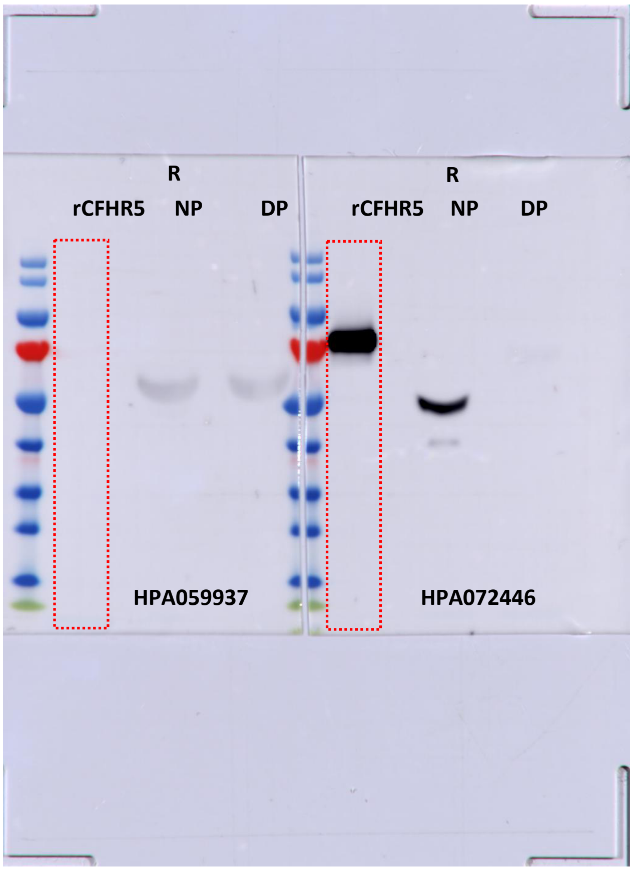

Supplement: Supplementary file 1 — Supplementary Information [file 41467_2023_38383_MOESM1_ESM.pdf]
