# Supplementary material for: Elevated plasma complement factor H related 5 protein is associated with venous thromboembolism
Source: Nat Commun. 2023 Jun 7;14:3280. doi: 10.1038/s41467-023-38383-y (PMC10247781; doi:10.1038/s41467-023-38383-y)
Supplement: Supplementary file 3 — Description of Additional Supplementary Files [file 41467_2023_38383_MOESM3_ESM.pdf]

**Description of Additional Supplementary Files**

**Supplementary Data 1**
Tab 1: Selection of antibodies with p values, FC and log P values (Citrate & EDTA)
Tab 2: Summary of the statistic values for Figure 1 c-i and 2 d-e
Tab 3: IC-MS SULF1
Tab 4: Risk factors & clinical chemistry
Tab 5: UAC
Tab 6: Liver co-expression
Tab 7: STRING
Tab 8: C3 and CFHR5
Tab 9: Meta sub-analyses
Tab 10: Sub-analysis (BMI, CRP)
Tab 11: GWAS metanalysis A
Tab 12: GWAS metanalysis B
Tab 13: GWAS metanalysis C
Tab 14: Rare variants
Tab 15: TGP analysis

**Supplementary Data 2**
Tab 1: VEBIOS Coagulation
Tab 2: DFW-VTE
Tab 3: FARIVE
Tab 4: RETROVE
Tab 5: MARTHA
Tab 6: ALL COHORTS
