# Supplementary material for: Elevated plasma complement factor H related 5 protein is associated with venous thromboembolism
Source: Nat Commun. 2023 Jun 7;14:3280. doi: 10.1038/s41467-023-38383-y (PMC10247781; doi:10.1038/s41467-023-38383-y)
Supplement: Supplementary file 6 — Reporting Summary [file 41467_2023_38383_MOESM6_ESM.pdf]

nature portfolio

Corresponding author(s): Jacob Odeberg
David Alexandre Tregouet

Last updated by author(s): Jan 9, 2023

Reporting Summary

Nature Portfolio wishes to improve the reproducibility of the work that we publish. This form provides structure for consistency and transparency in reporting. For further information on Nature Portfolio policies, see our Editorial Policies and the Editorial Policy Checklist.

Statistics

For all statistical analyses, confirm that the following items are present in the figure legend, table legend, main text, or Methods section.

n/a	Confirmed	
☐	☒	The exact sample size (*n*) for each experimental group/condition, given as a discrete number and unit of measurement
☐	☒	A statement on whether measurements were taken from distinct samples or whether the same sample was measured repeatedly
☐	☒	The statistical test(s) used AND whether they are one- or two-sided<br>*Only common tests should be described solely by name; describe more complex techniques in the Methods section.*
☐	☒	A description of all covariates tested
☐	☒	A description of any assumptions or corrections, such as tests of normality and adjustment for multiple comparisons
☐	☒	A full description of the statistical parameters including central tendency (e.g. means) or other basic estimates (e.g. regression coefficient) AND variation (e.g. standard deviation) or associated estimates of uncertainty (e.g. confidence intervals)
☐	☒	For null hypothesis testing, the test statistic (e.g. *F*, *t*, *r*) with confidence intervals, effect sizes, degrees of freedom and *P* value noted<br>*Give P values as exact values whenever suitable.*
☒	☐	For Bayesian analysis, information on the choice of priors and Markov chain Monte Carlo settings
☒	☐	For hierarchical and complex designs, identification of the appropriate level for tests and full reporting of outcomes
☐	☒	Estimates of effect sizes (e.g. Cohen's *d*, Pearson's *r*), indicating how they were calculated

Our web collection on statistics for biologists contains articles on many of the points above.

Software and code

Policy information about availability of computer code

Data collection	For collection of MFI values in proteomics screening and quantification using FlexMap 3D instrument (Luminex® Corp) and the instrument software xPONENT 4.3 was used. For identification of peptides in IC-MS, the search engine Sequest and Proteome Discoverer platform (PD, v1.4.0.339, Thermo Scientific) or MaxQuant (v. 2.1.4.0) . Peaks filter within ProteoWizard provided software tool msConvert (version 3.0.20321-6df943caa). Genotyping by Illumina Infinium Global Screening Array v2.0 and v3.0. Transcriptomics dataset were retrieved from Human Protein Atlas (v18-20) and Genotype-Tissue Expression (GTEx) Project (dbGaP Accession phs000424.v7.p2). Flow cytometry (Cytoflex, Beckman Coulter GmbH, Krefeld, Germany). Thrombinoscope software package (Version 3.0.0.29)
Data analysis	EncyclopeDIA (1.12.31) and Prosit (2018 model),integrated into ProteomicsDB (version 1.1) were employed for MS-DIA data. Eagle v2.4 & Minimac4 for imputation analyses ; Genesis & Plink1.9 and .2 for GWAS analyses; GWAMA (v2.2.2)) and METAL software (no version) for meta-analysis of GWAS results; Imputed genotypes by using MaCH version 1.0.18.c. WGCNA for regulatory gene network analysis. R versions from 3.2.0 to 4.0.5 (R Core Team 2021) for analysis of plasma proteomics and genetic data. GraphPad Prism for figures (version 9.1.2). Flow cytometry, data processed using Cytexpert v.2.5 (Beckman Coulter GmbH, Krefeld, Germany).

For manuscripts utilizing custom algorithms or software that are central to the research but not yet described in published literature, software must be made available to editors and reviewers. We strongly encourage code deposition in a community repository (e.g. GitHub). See the Nature Portfolio guidelines for submitting code & software for further information.

Data

Policy information about availability of data

All manuscripts must include a data availability statement. This statement should provide the following information, where applicable:

- Accession codes, unique identifiers, or web links for publicly available datasets
- A description of any restrictions on data availability
- For clinical datasets or third party data, please ensure that the statement adheres to our policy

DATA AVAILABILITY:
Source Data are provided with this paper.
The affinity proteomics data for VEBIOS ER generated in this study has been deposited in Figshare, https://doi.org/10.17044/scilifelab.22225942 [125]
The mass spectrometry proteomics data generated in this study has been deposited in the ProteomeXchange Consortium via the PRIDE [126] partner repository with the dataset identifier PXD040913. https://www.ebi.ac.uk/pride/archive/projects/PXD040913
The summary statistic of GWAS data generated in this study is available through GWAS catalogue (GCP ID: GCST90244658). https://www.ebi.ac.uk/gwas/studies/GCST90244658
For legal reasons and to minimize the possibility of unintentionally sharing information that can be used to re-identify private information, participant-level datasets containing full information (e.g. including sex, age, BMI, clinical data) cannot be openly shared. A subset of the data that support the findings of this study are available from the corresponding authors upon reasonable request (e.g. for validation). By contacting the corresponding authors (JO, DAT), procedures for sharing data, analytic methods, and study materials for reproducing the results or replicating the procedure can be arranged. When submitting an access request, please indicate: [name of PI and host organisation/contact details (including your name and email)/scientific purpose of data access request/commitment to inform when the data has been used in a publication/commitment not to host or share the data outside the requesting organisation/statement of non-commercial use of data].
External databases used:
Genotype-Tissue Expression (GTEx) Project (dbGaP Accession phs000424.v7.p2), www.gtexportal.org
Human Protein Atlas, human tissue expression data (v.18-20), www.proteinatlas.org
Homo Sapiens UniProt ID: #UP000009606; www.uniprot.org/proteomes/UP000009606
TOPMed r2 reference panel using Eagle v.2.4 (https://topmedimpute.readthedocs.io/en/latest/getting-started.html)
1000 Genomes phase 3 version reference panel (http://csg.sph.umich.edu/abecasis/mach/download/1000G.Phase3.v5.html
1000 Genomes Total European Ancestry (EUR) population (August 2010 release: http://csg.sph.umich.edu/abecasis/mach/download/1000G-2010-08.html; 1000G.EUR.20100804.tgz).

Human research participants

Policy information about studies involving human research participants and Sex and Gender in Research.

Reporting on sex and gender	Only data for biological sex has been used in the analyses and reported. All results are adjusted for sex and age. Sex stratified sub-analyses were performed in the different cohorts. Sex was considered in proteomics study design of VEBIOS ER and VEBIOS Coagulation in that cases and controls analysed were matched on sex and age. The sex of the individual anonymous blood donors from which platelets were isolated and used in functional studies was not considered in the analysis.
Population characteristics	VEBIOS ER: 48 cases/48 controls; Age 56.6 (19-89)years/56.6 (23-88) years; female sex 22/22; Smoking 4/9; family history VTE 14/6; Estrogens 5/5.<br><br>VEBIOS Coagulation: 144 cases/140 controls; Age 51.5(20-70)/53(22-71) years; Female sex 56/55; Smoking 22/19; family history VTE 24/1; Estrogens 26/15; Obesity 90/65.<br><br>DFW-VTE: 54 cases/146 controls; Age 64.8(20-96)/61.4(18-92); Female sex 23(43%)/90(62%); Estrogens 2/2.<br><br>FARIVE: 582 cases/576 controls; Age 53.1(17-91)/51.1(18-89); Female sex 348(60%)/330(57%); Smoking 95(16%)/137(24%); Obese 130 (22%)/109(19%); Estrogens 266/249.<br><br>RETROVE: 308 cases/360 controls); Age 55.4(17-79)/46(20-79); Female sex 142(46%)/183(51%); Obese 86(28%)/45(12.5%).<br><br>MARTHA: 774 cases; Age 46.7; Female sex 530(68%); Obese 96 (13%)
Recruitment	VEBIOS ER: Patients recruited in ER with suspected VTE. Biased towards patients with symptoms of VTE. Controls biased towards patients with medical ailment/symptoms prompting seeking medical care. This bias does not affect results as the analysis is aimed to finding diagnostic marker apliccable in this overall patient category. No bias between cases and controls as inclusion and blood sampling for biobanking is performed before diagnostic workup identifies cases and controls. VEBIOS ER study is a prospective cohort study carried out at the Emergency Room (ER) at the Karolinska University Hospital in Solna, Sweden, between December 2010 and September 2013. All patients admitted at ER with the suspicion of deep vein thrombosis (DVT) in the lower limbs and/or pulmonary embolism (PE), over 18 years old were eligible for the study.)<br><br>VEBIOS Coagulation: Patients recruited at outpatient clinic following ending treatment of VTE. Controls sex and age matched controls recruited from population. No apparent bias between cases and controls. VEBIOS Coagulation study is an on-going

case-control study established January 2011 of patients sampled at an outpatient coagulation clinic sampled 1-6 months after discontinuation of 6-12 months anticoagulant treatment after a verified first VTE (DVT to the lower limbs and/or PE), sex and age matched with healthy controls from the population. Patients were between 18 to 70 years of age, free from cancer, severe thrombophilia and pregnancy at inclusion.

DFW-VTE: Patients with suspected VTE recruited in ER. Biased towards patients with symptoms of VTE. Controls biased towards patients with ailment prompting seeking medical care. This bias does not affect results as analysis aimed to finding diagnostic marker in this overall patient category. (The Swedish Karolinska Age Adjusted D-Dimer study (DFW-VTE study) includes patients with clinically suspected acute VTE, prospectively recruited from the ER of Karolinska University Hospital in Huddinge, Stockholm. The patients were out-patients with low-to-high probability of acute PE or DVT in a lower limb.)

FARIVE: Patients recruited during initiation of treatment for VTE, with hospital based recruited controls. Controls biased towards patients with medical ailment resulting in hospital care. (The FARIVE study is a French multicentre case-control study carried out between 2003-2009. The study consists of patients with first confirmed VTE (DVT to the lower limbs and/or PE) from 18 years of age, matched to hospital controls with no previous thrombotic event).

RETROVE: Patients with VTE recruited after ending treatment. Controls recruited from population. No apparent bias. (The Riesgo de Enfermedad TROmboembólica VEnosa (RETROVE) study is a prospective case–control study of 400 consecutive patients with VTE (cancer associated thrombosis excluded) and 400 healthy control volunteers. Individuals were recruited at the Hospital de la Santa Creu i Sant Pau of Barcelona (Spain) between 2012 and 2016. Controls were selected according to the age and sex distribution of the Spanish population (2001 census).

MARTHA: Patients with prior VTE recruited and sampled at single center. No apparent bias. (The Marseille Thrombosis Association study (MARTHA) is a population based single centre study. Recruitment in MARTHA started in 1994 at Timone Hospital in Marseille (France) and is still ongoing. The cohort from 1994 and 2008, includes a total of 1542 VTE-cases (66% women) that donated blood for further analysis. All patients had a history of a first VTE documented by venography, Doppler ultrasound, angiography and/or ventilation/perfusion lung scan).

Ethics oversight

VEBIOS ER and VEBIOS Coagulation: regional research ethics committee in Stockholm, Sweden (KI 2010/636-31/4)
DFW-VTE: regional ethics review board in Stockholm (DNR 2013-2143-31-2).
FARIVE: Paris Broussais-HEGP ethics committee in Paris (2002-034)
RETROVE: Institutional Review Board of the Hospital de la Santa Creu i Sant Pau
MARTHA: Department of Health and Science, France (2008-880 & 09.576)
Platelet experiments: Human Ethics Committee of the Medical University of Vienna (EK237/2004)
HUVEC experiments: Regional Ethics Comitte Stockholm 2015/1552-32

Note that full information on the approval of the study protocol must also be provided in the manuscript.

Field-specific reporting

Please select the one below that is the best fit for your research. If you are not sure, read the appropriate sections before making your selection.

☒ Life sciences ☐ Behavioural & social sciences ☐ Ecological, evolutionary & environmental sciences

For a reference copy of the document with all sections, see nature.com/documents/nr-reporting-summary-flat.pdf

Life sciences study design

All studies must disclose on these points even when the disclosure is negative.

Sample size

VEBIOS ER: Nested case/control (48 cases/48 controls) derived from cohort of 158 patients with suspected acute VTE. Sample size determined by available number of confirmed cases.
VEBIOS Coagulation: case/control study (144 cases/140 controls). Sample size determined by available number of cases.
DFW-VTE cohort study: 200 patients (54 cases/146 controls). Sample size determined by number of patients included during set study period
FARIVE: Case/control study (582 cases/576 controls). Number determined based on available number of cases with VTE diagnosis.
MARTHA: Case-only cohort derived from case/control study: Size determined by number of patients with previous VTE for which follow-up data was available (669 patients), and for which data for TGP was available (774)
RETROVE:Case/control study (308 cases/360 controls).Sample size determined by number of patients included during set study period.

Data exclusions

Exclusion criteria in patient inclusion described below for respective study.
VEBIOS ER: preestablished exclusion criteria were patients with on-going anticoagulant treatment, pregnancy, active cancer, short life expectancy or lack of capacity to leave approved consent. No data generated from included patients were excluded.
VEBIOS Coagulation: cancer, severe thrombophilia and pregnancy at inclusion. No data from included patients were excluded.
DFW-VTE: No data exclusions.
FARIVE: No data from included patients were excluded.
RETROVE: No data exclusion for the patients in study set.

	MARTHA: Patients lacking followup data was excluded from analysis of recurrence. Patients lacking Thrombinoscope data was excluded from analysis of CFHR5 association with TGP.
Replication	The significant association signal for the antibody HPA059937 identified in the in the discovery single binder proteomics screen, identified by LC-MS to be CFHR5, was verified further with 3 different dual binder antibody assays in the discovery case/control set. An association with VTE was validated in the two discovery sets and 3 replication studies using a quantitative dual binder assay. All attempts at replication were successful.<br>Antibodies used in the quantitative dual binder assay were validated by Western blot and by Immunocapture-mass spectrometry.<br>Data independent acquisition mass spectrometry (DIA-MS) to perform orthogonal validation of the results obtained from the analysis of CFHR5 plasma levels in VEBIOS ER using the dual binder assay with capture antibody HPA072446, and monoclonal MAB3845.
Randomization	Randomization of samples was performed in the experimental set up using R, where samples were distributed in 96 well plates, balancing the number of case-control samples together with an average of age and sex numbers.
Blinding	We have and internal LIMS systems with new generated sample ID that does not track samples information during the experimental analyses. It was applied to all samples/cohorts analyzed. The origins of individual samples in the experimental set up were not know to the person performing the experiment.

Reporting for specific materials, systems and methods

We require information from authors about some types of materials, experimental systems and methods used in many studies. Here, indicate whether each material, system or method listed is relevant to your study. If you are not sure if a list item applies to your research, read the appropriate section before selecting a response.

Materials & experimental systems

n/a	Involved in the study	
☐	☒	Antibodies
☒	☐	Eukaryotic cell lines
☒	☐	Palaeontology and archaeology
☒	☐	Animals and other organisms
☐	☒	Clinical data
☒	☐	Dual use research of concern

Methods

n/a	Involved in the study	
☒	☐	ChIP-seq
☐	☒	Flow cytometry
☒	☐	MRI-based neuroimaging

Antibodies

Antibodies used

For dual binder validation of HPA059937 target ,anti-human CFHR5 (rabbit polyclonal HPA072446 and HPA073894), mouse anti-human CFHR5 (R&D systems, MAB3845, clone#390513) antibody

For CFHR5 absolute quantification in cohorts, rabbit polyclonal anti-human CFHR5 HPA072446 (Atlas Antibodies, 1,76ug/100ul buffer containing 0.5million magnetic beads) and mouse monoclonal anti-human CFHR5 (R&D systems; MAB3845, clone#390513, 1ug/ml)

For C3 quantification, mouse anti-human C3 and mouse monoclonal anti-human C3 antibodies (Bsi0263, Bsi0190, respectively, Biosystems International) were used in a dual binder assay

For C3c quantification, C3c ELISA kit cat. EKX-JD9XBE-96 (Nordic BioSite, Sweden). Specificity of the assay provided by the company:
Vendor statement: This assay has high sensitivity and excellent specificity for detection of C3c. No significant cross-reactivity or interference between C3c and analogues was observed. Note: Limited by current skills and knowledge, it is difficult for us to complete the cross-reactivity detection between C3c and all the analogues, therefore, cross reaction may still exist.

For platelet assays: anti-human CD62P-AF647 (clone#AK4, cat.304918), anti-human CD63-PE (clone#H5C6, cat.353004) or anti-human CD41/CD61-FITC (clone:PAC-1, cat.362804) (all Biolegend)

For proteomics screening: (Table S1, Tab 1. on the main manuscript) - [1,76ug/100ul buffer containing 0.5 million magnetic beads]

HPA Antibody UniProt Protein symbol
HPA059937 Q8IWU6 SULF1
HPA044659 Q08722 CD47
HPA002655 P16109 SELP
HPA003042 P29274 ADORA2A
HPA037423 P30530 AXL
HPA050269 P04196 HRG
HPA001616 P01042 KNG1
HPA046773 Q8TDI8 TMC1
HPA002082 P04275 VWF
HPA026290 P20851 C4BPB

HPA053470 Q30201 HFE
HPA047725 P02763;P19652 ORM1;ORM2
HPA042212 A4FU49 SH3D21
HPA011972 P01127 PDGFB
HPA050724 P15822 HIVEP1
HPA059686 P22352 GPX3
HPA000594 P00451 F8
HPA001815 P04275 VWF
HPA007384 P05160 F13B
HPA035199 O15530 PDPK1
HPA036058 Q86TI2 DPP9
HPA048862 Q9BXT6 MOV10L1
HPA056729 P78423 CX3CL1
HPA064215 Q9UPY5 SLC7A11
HPA006225 P16581 SELE
HPA013149 Q01082 SPTBN1
HPA024190 P36871 PGM1
HPA036660 Q9NQ30 ESM1
HPA039991 Q5T5C0 STXBP5
HPA046055 P20382 PMCH
HPA047992 Q8N699 MYCT1
HPA048154 Q9HCC8 GDPD2
HPA049741 Q5SY16 NOL9
HPA050823 Q9H5Z6 FAM124B
HPA052555 Q6UX40 TMEM107
HPA052957 Q16549 PCSK7
HPA055126 Q6UVM3 KCNT2
HPA055461 P49767 VEGFC
HPA057242 Q14247 CTTN
HPA064606 P01584 IL1B
HPA031471 P45844 ABCG1
HPA001866 P42684 ABL2
HPA017672 Q16570 ACKR1
HPA031498 Q9UHI8 ADAMTS1
HPA042014 Q76LX8 ADAMTS13
HPA052077 Q76LX8 ADAMTS13
HPA044326 Q8TE60 ADAMTS18
HPA015658 O60242 ADGRB3
HPA015963 O60242 ADGRB3
HPA025229 Q9HBW9 ADGRL4
HPA011323 P29274 ADORA2A
HPA011970 P29274 ADORA2A
HPA065566 P29274 ADORA2A
HPA048578 P08913 ADRA2A
HPA066264 P08913 ADRA2A
HPA003431 P07550 ADRB2
HPA030153 Q8WV93 AFG1L
HPA030154 Q8WV93 AFG1L
HPA030155 Q8WV93 AFG1L
HPA037382 Q9BYV1 AGXT2
HPA006171 Q96IF1 AJUBA
HPA055116 Q96AK3 AL022318.4;APOBEC3D
HPA049508 O15123 ANGPT2
HPA064988 O15123 ANGPT2
HPA045916 Q15327 ANKRD1
HPA036276 Q9NQ90 ANO2
HPA036277 Q9NQ90 ANO2
HPA038958 Q4KMQ2 ANO6
HPA058737 Q4KMQ2 ANO6
HPA046964 P07355 ANXA2
HPA061798 P07355 ANXA2
HPA046715 P02647 APOA1
HPA001352 P06727 APOA4
HPA002549 P06727 APOA4
HPA005149 P06727 APOA4
HPA039229 Q6Q788 APOA5
HPA049956 Q6Q788 APOA5
HPA063565 Q6Q788 APOA5

HPA049793 P04114 APOB
HPA055096 P04114 APOB
HPA041667 Q0VD83 APOBR
HPA042093 Q0VD83 APOBR
HPA056395 P02656 APOC3
HPA065365 P02656 APOC3
HPA040520 P05090 APOD
HPA065539 P02649 APOE
HPA063325 Q13790 APOF
HPA001654 P02749 APOH
HPA003732 P02749 APOH
HPA044180 O95445 APOM
HPA051006 O95445 APOM
HPA056226 O95445 APOM
HPA026534 Q52LW3 ARHGAP29
HPA059074 Q8WYA1 ARNTL2
HPA065355 Q8WYA1 ARNTL2
HPA036481 Q5FYB0 ARSJ
HPA051148 P52961 ART1
HPA040879 Q93070 ART4
HPA038444 O95260 ATE1
HPA055069 P18846 ATF1
HPA055406 P18846 ATF1
HPA037422 P30530 AXL
HPA040081 P54687 BCAT1
HPA048592 P54687 BCAT1
HPA059106 P54687 BCAT1
HPA056104 P23560 BDNF
HPA054235 P51451 BLK
HPA005711 Q9H2G9 BLZF1
HPA025703 Q9H2G9 BLZF1
HPA027331 Q9H2G9 BLZF1
HPA067113 Q9H2G9 BLZF1
HPA058610 P12643 BMP2
HPA059913 P12643 BMP2
HPA041077 P12644 BMP4
HPA066235 P12644 BMP4
HPA062683 P22004 BMP6
HPA068915 P22004 BMP6
HPA000512 P51813 BMX
HPA001048 P51813 BMX
HPA045739 Q5SWW7 C10orf55
HPA055347 Q8N8G6 C15orf54
HPA028452 Q8NDD1 C1orf131
HPA029920 Q8NDD1 C1orf131
HPA050076 Q8N268 C20orf197
HPA046432 Q3SXR2 C3orf36
HPA000926 P04003 C4BPA
HPA001578 P04003 C4BPA
HPA001845 P04003 C4BPA
HPA051620 P20851 C4BPB
HPA062357 P20851 C4BPB
HPA051506 Q9BY67 CADM1
HPA007586 Q16602 CALCRL
HPA034969 Q8N5C1 CALHM5
HPA034970 Q8N5C1 CALHM5
HPA055419 Q8N5C1 CALHM5
HPA042524 Q96M63 CCDC114
HPA062146 Q96M63 CCDC114
HPA028584 Q86UF4 CCDC190
HPA028592 Q86UF4 CCDC190
HPA042909 O15467 CCL16
HPA051577 O15467 CCL16
HPA068091 O15467 CCL16
HPA040876 Q92583 CCL17
HPA058037 Q92583 CCL17
HPA010552 P13501 CCL5
HPA045228 P80098 CCL7

HPA026934 P32246 CCR1
HPA065477 P32246 CCR1
HPA044929 P51677 CCR3
HPA008992 Q6YHK3 CD109
HPA002839 Q9NZQ7 CD274
HPA003528 Q9NZQ7 CD274
HPA005484 Q9NZQ7 CD274
HPA063154 Q9NZQ7 CD274
HPA001007 P29965 CD40LG
HPA001371 P29965 CD40LG
HPA045827 P29965 CD40LG
HPA044659 Q08722 CD47
HPA010088 P08962 CD63
HPA041454 Q01151 CD83
HPA043887 Q01151 CD83
HPA005677 P21926 CD9
HPA009000 Q9NPY3 CD93
HPA009300 Q9NPY3 CD93
HPA039906 Q86UT8 CENATAC
HPA023430 Q53EZ4 CEP55
HPA053078 Q53EZ4 CEP55
HPA038275 Q70JA7 CHSY3
HPA044612 Q70JA7 CHSY3
HPA039468 Q86T13 CLEC14A
HPA066399 Q86T13 CLEC14A
HPA035671 Q7Z7G1 CLNK
HPA035672 Q7Z7G1 CLNK
HPA035149 P27658 COL8A1
HPA053107 P27658 COL8A1
HPA004146 Q96IY4 CPB2
HPA048375 Q8N436 CPXM2
HPA065096 Q8N436 CPXM2
HPA007688 Q9NQ79 CRTAC1
HPA008175 Q9NQ79 CRTAC1
HPA030948 Q9Y4K1 CRYBG1
HPA030949 Q9Y4K1 CRYBG1
HPA039288 P04141 CSF2
HPA048058 P04141 CSF2
HPA057404 P04141 CSF2
HPA001412 P09919 CSF3
HPA007301 Q9P2B4 CTTNBP2NL
HPA007328 Q9P2B4 CTTNBP2NL
HPA040361 P78423 CX3CL1
HPA046579 P02778 CXCL10
HPA054954 P02778 CXCL10
HPA048978 P48061 CXCL12
HPA065474 P42830 CXCL5
HPA048879 P80162 CXCL6
HPA045822 P10145 CXCL8
HPA057179 P10145 CXCL8
HPA031991 P25024 CXCR1
HPA031993 P25024 CXCR1
HPA064353 P25024 CXCR1
HPA031999 P25025 CXCR2
HPA032016 P25025 CXCR2
HPA008401 Q9NS75 CYSLTR2
HPA008605 Q9NS75 CYSLTR2
HPA046528 Q9NS75 CYSLTR2
HPA021072 Q5VWQ8 DAB2IP
HPA036977 Q5VWQ8 DAB2IP
HPA017167 P52429 DGKE
HPA004917 Q9NSV4 DIAPH3
HPA032152 Q9NSV4 DIAPH3
HPA002806 Q9H7Y0 DIPK2B
HPA054105 Q9Y2H0 DLGAP4
HPA056378 Q9Y2H0 DLGAP4
HPA031976 P05305 EDN1
HPA055622 P05305 EDN1

HPA068325 P05305 EDN1
HPA023109 Q8N7B9 EFCAB3
HPA023317 Q8N7B9 EFCAB3
HPA030717 Q8N7B9 EFCAB3
HPA046862 Q8N7B9 EFCAB3
HPA062231 Q12805 EFEMP1
HPA035307 Q5JVL4 EFHC1
HPA043442 Q5JVL4 EFHC1
HPA066836 P08246 ELANE
HPA027572 P01588 EPO
HPA019237 Q9UBC2 EPS15L1
HPA055309 Q9UBC2 EPS15L1
HPA003275 P11308 ERG
HPA046598 P11308 ERG
HPA039363 P30040 ERP29
HPA063781 Q9BS26 ERP44
HPA005787 Q9NQ30 ESM1
HPA015110 P58658 EVA1C
HPA015720 P58658 EVA1C
HPA029944 P58658 EVA1C
HPA029945 P58658 EVA1C
HPA059525 P00742 F10
HPA063273 P00742 F10
HPA056201 P03951 F11
HPA058830 P03951 F11
HPA067405 P03951 F11
HPA067557 P03951 F11
HPA043616 Q9Y624 F11R
HPA061700 Q9Y624 F11R
HPA001597 P00488 F13A1
HPA001804 P00488 F13A1
HPA047903 P00488 F13A1
HPA003827 P05160 F13B
HPA027563 P05160 F13B
HPA052139 P05160 F13B
HPA051476 P00734 F2
HPA054698 P00734 F2
HPA049045 P25116 F2R
HPA052785 P55085 F2RL1
HPA019328 Q96RI0 F2RL3
HPA031401 Q96RI0 F2RL3
HPA042340 Q96RI0 F2RL3
HPA058724 Q96RI0 F2RL3
HPA049292 P13726 F3
HPA002036 P12259 F5
HPA005942 P12259 F5
HPA050035 P12259 F5
HPA063808 P08709 F7
HPA000284 P00451 F8
HPA042729 Q9H5Z6 FAM124B
HPA048345 Q8N2R8 FAM43A
HPA064589 Q8N2R8 FAM43A
HPA056898 Q6V0I7 FAT4
HPA025287 Q8WUP2 FBLIM1
HPA008928 P06734 FCER2
HPA017162 P06734 FCER2
HPA067430 P06734 FCER2
HPA010718 P12318 FCGR2A
HPA010776 P12318 FCGR2A
HPA051370 P02671 FGA
HPA064755 P02671 FGA
HPA065502 P09038 FGF2
HPA019229 Q14314 FGL2
HPA055365 Q01543 FLI1
HPA065030 Q01543 FLI1
HPA065261 Q01543 FLI1
HPA062635 O43155 FLRT2
HPA047848 Q99958 FOXC2

HPA065354 Q99958 FOXC2
HPA011650 Q7Z6J6 FRMD5
HPA011746 Q7Z6J6 FRMD5
HPA041602 P02792 FTL
HPA051551 Q8N3T1 GALNT15
HPA056080 Q14393 GAS6
HPA000232 P15976 GATA1
HPA000233 P15976 GATA1
HPA000890 P15976 GATA1
HPA018468 Q9NR23 GDF3
HPA045206 Q6KF10 GDF6
HPA051822 Q7Z4P5 GDF7
HPA045026 Q9HCC8 GDPD2
HPA047675 Q03113 GNA12
HPA054229 Q03113 GNA12
HPA010087 Q14344 GNA13
HPA050978 P30679 GNA15
HPA018122 O95467;P63092;P84996;Q5JWF2 GNAS
HPA027479 O95467;P63092;P84996;Q5JWF2 GNAS
HPA028386 O95467;P63092;P84996;Q5JWF2 GNAS
HPA048381 P40197 GP5
HPA064082 P40197 GP5
HPA065071 P40197 GP5
HPA048214 Q9HCN6 GP6
HPA060965 Q9HCN6 GP6
HPA066482 Q9HCN6 GP6
HPA054665 P14770 GP9
HPA063182 P14770 GP9
HPA039943 Q14439 GPR176
HPA039979 Q14439 GPR176
HPA013783 P32249 GPR183
HPA013784 P32249 GPR183
HPA028847 P32249 GPR183
HPA047847 P22352 GPX3
HPA062579 P22352 GPX3
HPA067055 P22352 GPX3
HPA036720 Q8TED1 GPX8
HPA036721 Q8TED1 GPX8
HPA042431 O15544 GR6 / C3orf27
HPA023670 Q9BYG8 GSDMC
HPA026317 Q9BYG8 GSDMC
HPA021074 Q14520 HABP2
HPA037539 P55084 HADHB
HPA037540 P55084 HADHB
HPA066099 P55084 HADHB
HPA045254 P81172 HAMP
HPA065275 P81172 HAMP
HPA052080 Q92839 HAS1
HPA060273 Q92839 HAS1
HPA067602 Q92839 HAS1
HPA051563 Q96QV1 HHIP
HPA067457 P15822 HIVEP1
HPA051677 Q96RW7 HMCN1
HPA039076 P52926 HMGA2
HPA056516 P31942 HNRNPH3
HPA061982 P31269 HOXA9
HPA066795 P00738 HP
HPA054598 P04196 HRG
HPA028808 P28222 HTR1B
HPA055270 P28222 HTR1B
HPA014011 P28223 HTR2A
HPA064731 P28223 HTR2A
HPA052903 P28335 HTR2C
HPA051787 Q4G0P3 HYDIN
HPA067155 Q4G0P3 HYDIN
HPA049525 P01579 IFNG
HPA053530 P01579 IFNG
HPA063125 P01579 IFNG

HPA048946 P05019 IGF1
HPA052648 P05019 IGF1
HPA004822 P08069 IGF1R
HPA045563 P08069 IGF1R
HPA052949 P08069 IGF1R
HPA062273 Q9NZI8 IGF2BP1
HPA035145 Q9Y6M1 IGF2BP2
HPA054591 A1L1A6 IGSF23
HPA051182 P22301 IL10
HPA063270 P22301 IL10
HPA044955 P20809 IL11
HPA059191 P20809 IL11
HPA048546 P01583 IL1A
HPA044649 P01584 IL1B
HPA007917 Q01638 IL1RL1
HPA008412 P08700 IL3
HPA030770 P08700 IL3
HPA066598 P08700 IL3
HPA040444 P24394 IL4R
HPA044116 P24394 IL4R
HPA050124 P24394 IL4R
HPA059876 P24394 IL4R
HPA058514 A6NMK8 INSYN2B
HPA050862 Q9H1B7 IRF2BPL
HPA061333 Q9H1B7 IRF2BPL
HPA046433 P35568 IRS1
HPA050221 P35568 IRS1
HPA045721 Q9Y4H2 IRS2
HPA046618 O75578 ITGA10
HPA064008 O75578 ITGA10
HPA031169 P08514 ITGA2B
HPA031170 P08514 ITGA2B
HPA031171 P08514 ITGA2B
HPA042950 P13612 ITGA4
HPA052415 P13612 ITGA4
HPA064041 P13612 ITGA4
HPA002642 P08648 ITGA5
HPA003520 P08648 ITGA5
HPA012696 P23229 ITGA6
HPA027582 P23229 ITGA6
HPA053298 P20701 ITGAL
HPA002274 P11215 ITGAM
HPA051326 P05556 ITGB1
HPA064704 Q14573 ITPR3
HPA003417 Q9BX67 JAM3
HPA051218 Q6UVM3 KCNT2
HPA024205 Q86Y91 KIF18B
HPA027831 Q86Y91 KIF18B
HPA028695 Q86Y91 KIF18B
HPA068315 Q86Y91 KIF18B
HPA036909 O95235 KIF20A
HPA036910 O95235 KIF20A
HPA066080 O95235 KIF20A
HPA061862 P21583 KITLG
HPA053837 Q9P2N7 KLHL13
HPA035237 Q96CT2 KLHL29
HPA057379 Q96CT2 KLHL29
HPA001646 P01042 KNG1
HPA015141 Q16363 LAMA4
HPA015693 Q16363 LAMA4
HPA048033 Q16363 LAMA4
HPA014750 P11279 LAMP1
HPA054704 P11279 LAMP1
HPA029100 P13473 LAMP2
HPA044767 P04180 LCAT
HPA038250 Q86YD5 LDLRAD3
HPA038251 Q86YD5 LDLRAD3
HPA030721 P41159 LEP

HPA030722 P41159 LEP
HPA057322 P41159 LEP
HPA030898 P48357 LEPR
HPA030899 P48357 LEPR
HPA018844 P15018 LIF
HPA029950 Q6XZB0 LIPI
HPA049665 Q6XZB0 LIPI
HPA006660 P02545 LMNA
HPA060604 P08519 LPA
HPA063726 P08519 LPA
HPA006224 Q92633 LPAR1
HPA050667 Q92633 LPAR1
HPA048749 P06858 LPL
HPA066857 P06858 LPL
HPA004182 Q07954 LRP1
HPA064792 P98164 LRP2
HPA015671 Q14114 LRP8
HPA051347 Q14114 LRP8
HPA063469 Q14114 LRP8
HPA064106 Q14114 LRP8
HPA013409 Q7Z2Q7 LRRC70
HPA042061 Q8N2G4 LYPD1
HPA043594 Q8N2G4 LYPD1
HPA068335 Q8N2G4 LYPD1
HPA053561 Q86Y78 LYPD6
HPA043434 Q96GV9 MACIR
HPA063825 Q9P2E8 MARCHF4
HPA008848 P43121 MCAM
HPA020038 P43121 MCAM
HPA063834 P43121 MCAM
HPA053763 Q5VYS4 MEDAG
HPA036195 Q12866 MERTK
HPA036196 Q12866 MERTK
HPA054688 P03956 MMP1
HPA066763 P03956 MMP1
HPA052343 P09238 MMP10
HPA053433 P09238 MMP10
HPA036107 P45452 MMP13
HPA054569 P45452 MMP13
HPA059580 P45452 MMP13
HPA065038 P08254 MMP3
HPA063909 P14780 MMP9
HPA058108 Q13201 MMRN1
HPA007619 P40238 MPL
HPA067472 P40238 MPL
HPA061464 P05164 MPO
HPA036387 Q96JB8 MPP4
HPA057393 Q96JB8 MPP4
HPA008966 Q96G30 MRAP2
HPA009039 Q96G30 MRAP2
HPA017642 Q9HD23 MRS2
HPA052000 Q9HD23 MRS2
HPA055174 Q1L6U9 MSMP
HPA062800 Q1L6U9 MSMP
HPA029040 Q6UB35 MTHFD1L
HPA029041 Q6UB35 MTHFD1L
HPA029042 Q6UB35 MTHFD1L
HPA020660 P42898 MTHFR
HPA063389 P42898 MTHFR
HPA021515 Q8N699 MYCT1
HPA047091 A7E2Y1 MYH7B
HPA052679 A7E2Y1 MYH7B
HPA056262 A7E2Y1 MYH7B
HPA064516 A7E2Y1 MYH7B
HPA018128 Q15746 MYLK
HPA031677 Q15746 MYLK
HPA048055 Q15746 MYLK
HPA057158 Q9Y3Q0 NAALAD2

HPA060820 Q9Y3Q0 NAALAD2
HPA065419 Q9Y3Q0 NAALAD2
HPA042411 Q09161 NCBP1
HPA049031 Q09161 NCBP1
HPA039883 P46934 NEDD4
HPA046793 P46934 NEDD4
HPA026763 Q9UMX5 NENF
HPA028488 Q9UMX5 NENF
HPA001914 Q16621 NFE2
HPA049771 Q5HYW2 NHSL2
HPA054858 Q5HYW2 NHSL2
HPA059180 P40261 NNMT
HPA065727 P40261 NNMT
HPA046122 Q5SY16 NOL9
HPA054986 Q5SY16 NOL9
HPA063926 Q5SY16 NOL9
HPA010964 Q02297 NRG1
HPA038206 P56975 NRG3
HPA038207 P56975 NRG3
HPA017357 P21589 NT5E
HPA017363 P21589 NT5E
HPA048043 P21589 NT5E
HPA049832 Q9HB63 NTN4
HPA027455 O60285 NUAK1
HPA027460 O60285 NUAK1
HPA027462 O60285 NUAK1
HPA057143 O60285 NUAK1
HPA062351 Q9H1E3 NUCKS1
HPA042761 A8MXV4 NUDT19
HPA053125 A8MXV4 NUDT19
HPA035619 P78380 OLR1
HPA035620 P78380 OLR1
HPA050798 P78380 OLR1
HPA054772 P78380 OLR1
HPA047127 O76099 OR7C1
HPA029814 P13725 OSM
HPA040397 P51575 P2RX1
HPA044205 P51575 P2RX1
HPA059917 P51575 P2RX1
HPA015577 P47900 P2RY1
HPA007601 Q7Z4N8 P4HA3
HPA018884 P07237 P4HB
HPA017959 Q9NP74 PALMD
HPA030549 Q9NP74 PALMD
HPA011835 Q9P2E7 PCDH10
HPA011905 Q8WUM4 PDCD6IP
HPA051110 Q8WUM4 PDCD6IP
HPA051604 Q8WUM4 PDCD6IP
HPA045273 P04085 PDGFA
HPA051985 P04085 PDGFA
HPA063058 P04085 PDGFA
HPA065024 P04085 PDGFA
HPA011325 P01127 PDGFB
HPA006139 P13667 PDIA4
HPA006337 P13667 PDIA4
HPA034652 Q15084 PDIA6
HPA034653 Q15084 PDIA6
HPA036503 O15018 PDZD2
HPA036504 O15018 PDZD2
HPA064387 O15018 PDZD2
HPA024637 P36871 PGM1
HPA018018 Q6PCE3 PGM2L1
HPA056995 Q6PCE3 PGM2L1
HPA004100 P42338 PIK3CB
HPA028903 P42338 PIK3CB
HPA064207 P42338 PIK3CB
HPA001743 O00625 PIR
HPA041873 Q9UP65 PLA2G4C

HPA043083 Q9UP65 PLA2G4C
HPA053548 Q9UP65 PLA2G4C
HPA048823 P00747 PLG
HPA045188 Q5VZY2 PLPP4
HPA052141 Q9HCM2 PLXNA4
HPA040852 O15305 PMM2
HPA063649 O15305 PMM2
HPA059037 Q01851 POU4F1
HPA068024 Q01851 POU4F1
HPA047248 Q96QC0 PPP1R10
HPA056756 Q96QC0 PPP1R10
HPA045286 O95522 PRAMEF12
HPA001235 Q96MT3 PRICKLE1
HPA050037 Q96MT3 PRICKLE1
HPA005550 P04070 PROC
HPA050030 P04070 PROC
HPA039461 Q9UNN8 PROCR
HPA042831 Q9UNN8 PROCR
HPA023974 P07225 PROS1
HPA016503 P22891 PROZ
HPA052006 P22891 PROZ
HPA042924 Q9Y617 PSAT1
HPA051588 Q9Y617 PSAT1
HPA048972 O43242 PSMD3
HPA035381 Q6ZW05 PTCHD4
HPA041276 Q6ZW05 PTCHD4
HPA054984 Q6ZW05 PTCHD4
HPA029829 P43119 PTGIR
HPA046574 P43119 PTGIR
HPA049663 P43119 PTGIR
HPA066204 P43119 PTGIR
HPA038342 P26045 PTPN3
HPA038343 P26045 PTPN3
HPA046728 P26045 PTPN3
HPA054453 P23467 PTPRB
HPA067868 P23467 PTPRB
HPA065722 P26022 PTX3
HPA044920 Q53H96 PYCR3
HPA053776 Q53H96 PYCR3
HPA063416 Q53H96 PYCR3
HPA061377 Q96E17 RAB3C
HPA036950 Q9P0K7 RAI14
HPA015667 Q7LDG7 RASGRP2
HPA045885 P57052 RBM11
HPA065792 P57052 RBM11
HPA048969 P48378 RFX2
HPA047942 P61587 RND3
HPA055364 P61587 RND3
HPA065881 P61587 RND3
HPA007567 Q13464 ROCK1
HPA008004 Q13464 ROCK1
HPA026828 P04843 RPN1
HPA037912 Q01196 RUNX1
HPA049852 Q06455 RUNX1T1
HPA053290 Q06455 RUNX1T1
HPA064531 Q06455 RUNX1T1
HPA066761 Q06455 RUNX1T1
HPA052603 Q9NZJ4 SACS
HPA048488 Q9UPU9 SAMD4A
HPA065309 Q9UPU9 SAMD4A
HPA049515 Q8WTV0 SCARB1
HPA054712 Q8WTV0 SCARB1
HPA043937 Q9UQD0 SCN8A
HPA058780 Q9UQD0 SCN8A
HPA064422 Q9UQD0 SCN8A
HPA040196 P53992 SEC24C
HPA040213 P53992 SEC24C
HPA027227 P16581 SELE

HPA057891 P16581 SELE
HPA065850 P16581 SELE
HPA011902 Q14242 SELPLG
HPA022802 Q14242 SELPLG
HPA044890 Q14242 SELPLG
HPA052704 Q14242 SELPLG
HPA048739 Q9UK55 SERPINA10
HPA061957 P05154 SERPINA5
HPA055767 P05546 SERPIND1
HPA024527 P05121 SERPINE1
HPA045409 P05121 SERPINE1
HPA048738 P05155 SERPING1
HPA048105 Q9BWM7 SFXN3
HPA042456 A4FU49 SH3D21
HPA066168 A4FU49 SH3D21
HPA045205 P04278 SHBG
HPA053902 A0PJX4 SHISA3
HPA010089 Q9ULL8 SHROOM4
HPA010565 Q9ULL8 SHROOM4
HPA039040 Q9H6Q3 SLA2
HPA046136 Q9H6Q3 SLA2
HPA053746 Q9H6Q3 SLA2
HPA066826 Q9H6Q3 SLA2
HPA064728 Q8TBE7 SLC35G2
HPA031532 Q8N695 SLC5A8
HPA053103 Q8N695 SLC5A8
HPA042110 P08294 SOD3
HPA012378 Q92673 SORL1
HPA031320 Q92673 SORL1
HPA031321 Q92673 SORL1
HPA031322 Q92673 SORL1
HPA006889 Q99523 SORT1
HPA037884 Q9BXB7 SPATA16
HPA038441 Q9BXB7 SPATA16
HPA005628 P17947 SPI1
HPA044653 P17947 SPI1
HPA030875 P12931 SRC
HPA058020 O60687 SRPX2
HPA019845 Q8WYL5 SSH1
HPA052749 Q8WYL5 SSH1
HPA023463 Q76I76 SSH2
HPA044386 Q76I76 SSH2
HPA057099 Q76I76 SSH2
HPA054566 Q8NDV1 ST6GALNAC3
HPA026871 Q8WWQ8 STAB2
HPA038189 P32856 STX2
HPA057594 P32856 STX2
HPA001330 Q12846 STX4
HPA063868 Q15833 STXBP2
HPA030958 Q5T5C0 STXBP5
HPA049727 Q5T5C0 STXBP5
HPA003552 Q8NFX7 STXBP6
HPA029300 Q8NFX7 STXBP6
HPA051204 Q8IWU6 SULF1
HPA054728 Q8IWU6 SULF1
HPA028728 P49888 SULT1E1
HPA001384 P43405 SYK
HPA047866 P17542 TAL1
HPA017082 P21731 TBXA2R
HPA051183 P21731 TBXA2R
HPA065207 P21731 TBXA2R
HPA065250 P21731 TBXA2R
HPA031257 P24557 TBXAS1
HPA031258 P24557 TBXAS1
HPA031259 P24557 TBXAS1
HPA031260 P24557 TBXAS1
HPA027549 Q8N9U0 TC2N
HPA041631 P20061 TCN1

HPA043905 P20061 TCN1
HPA005575 P10646 TFPI
HPA011937 Q9UP52 TFR2
HPA017171 Q9UP52 TFR2
HPA047516 P01137 TGFB1
HPA049818 P61812 TGFB2
HPA060541 P61812 TGFB2
HPA065065 P61812 TGFB2
HPA067580 P61812 TGFB2
HPA037420 Q9H5L6 THAP9
HPA037421 Q9H5L6 THAP9
HPA021513 P07996 THBS1
HPA042942 P07996 THBS1
HPA019596 P40225 THPO
HPA042965 P40225 THPO
HPA048828 P40225 THPO
HPA051629 P40225 THPO
HPA053417 P01033 TIMP1
HPA064888 P16035 TIMP2
HPA051828 Q9Y4G6 TLN2
HPA054787 Q9Y4G6 TLN2
HPA056857 Q9BZW5 TM6SF1
HPA044166 Q8TDI8 TMC1
HPA028736 Q8N7C4 TMEM217
HPA045432 Q8N7C4 TMEM217
HPA059261 Q8N7C4 TMEM217
HPA041921 Q2M3C6 TMEM266
HPA041977 Q2M3C6 TMEM266
HPA049425 Q2M3C6 TMEM266
HPA050631 P01375 TNF
HPA055037 P01375 TNF
HPA064998 P01375 TNF
HPA037513 Q9UBN6 TNFRSF10D
HPA055993 Q9UBN6 TNFRSF10D
HPA065387 Q9UBN6 TNFRSF10D
HPA016700 Q86YW5 TREML1
HPA017860 Q86YW5 TREML1
HPA023980 Q9UJT1 TUBD1
HPA027090 Q9UJT1 TUBD1
HPA043871 Q16881 TXNRD1
HPA062986 Q06418 TYRO3
HPA041943 P26368 U2AF2
HPA064409 P26368 U2AF2
HPA038839 Q8IWV7 UBR1
HPA046727 Q8IWV7 UBR1
HPA038195 Q6UW78 UQCC3
HPA046851 Q6UW78 UQCC3
HPA036233 P22415 USF1
HPA036535 P22415 USF1
HPA006882 Q9BV40 VAMP8
HPA007215 Q9BV40 VAMP8
HPA063777 P18206 VCL
HPA053984 P15692 VEGFA
HPA063302 P15692 VEGFA
HPA055171 P49767 VEGFC
HPA026645 Q14D04 VEPH1
HPA041588 Q14D04 VEPH1
HPA051312 P98155 VLDLR
HPA002022 P42768 WAS
HPA039090 Q9UPY6 WASF3
HPA003739 O43516 WIPF1
HPA042428 P98170 XIAP
HPA055105 B2RXF5 ZBTB42
HPA066961 B2RXF5 ZBTB42
HPA023128 Q8N895 ZNF366
HPA023526 Q8N895 ZNF366
HPA052024 Q96IT1 ZNF496
HPA055834 Q96IT1 ZNF496

Western blot:
horseradish peroxidase (HRP)-coupled goat anti-rabbit (cat:P0448) or anti-mouse antibodies (cat:P0447)(both, 1:2000, Dako)

Validation

The target of antibody HPA059937 was validated by immunocapture Mass Spectrometry (IC-MS), and results in the discovery single binder proteomics screen was further validated by 3 different dual binder antibody assay, with correlation values >0.7, as descibed in the manuscript.

The HPA072446, targeting the CFHR5 protein, was validated iby 2 dual binder assays and by western blot recognizing correctly the recombinant CFHR5 as described in manuscript. Antibody capture of CFHR5 in plasma was validated by IC-MS.

The monoclonal MAB3845 (R&D) was validated by the vendor (vendor statement: "antibody has been validated for the following applications: Western Blot, Immunoprecipitation") and independently validated in house by IC-MS and Western blot recognizing correctly the recombinant CFHR5, as described in manuscript.

The Dual binder assay for CFHR5 composed of HPA072446 and MAB3845 was validated by correlating with data obtained through Data Independent Aquisition Mass Spectropmetry (DIA-MS) in 96 samples of VEBIOS ER cohort which it is obtained independently of the use of affinity reagents such as antibodies (described in manuscript).

The ELISA kit for C3c was validated by the vendor (Nordic BioSite, cat:EKX-JD9XBE-96). (Vendor statement: "This assay has high sensitivity and excellent specificity for detection of C3c. No significant cross-reactivity or interference between C3c and analogues was observed. Note: Limited by current skills and knowledge, it is difficult for us to complete the cross-reactivity detection between C3c and all the analogues, therefore, cross reaction may still exist").

Validation data for HPA antibodies was obtained from www.proteinatlas.org. (/www.proteinatlas.org/about/antibody+validation)

All antibodies produced internally within the Human Protein Atlas project (HPA antibodies) must pass steps 1-4 in the list below in order to be used for immunohistochemistry and immunocytochemistry/IF. Steps 5-7 provide the basis for evaluating and scoring the antibody reliability. All antibodies that provide a reasonable pattern of immunoreactivity are added to the Human Protein Atlas portal. Feedback from the research community is appreciated and needed for continuous curation of data.

Quality assurance steps for antibodies generated within the Human Protein Atlas project:

The antigen (protein epitope signature tag (PrEST) for a protein is selected as a stretch of 20-150 amino acids with as low identity as possible to proteins from all other putative protein-coding genes, and not including signal peptides or transmembrane regions. Multitarget PrESTs are PrESTs that have more than 80% identity to proteins from more than one gene, and are expected to generate antibodies with multiple targets.

Plasmid inserts are sequenced to assure that the correct PrEST sequence is cloned.

Size of the resulting recombinant protein (including the specific PrEST) is analyzed using mass spectrometry to assure that the correct antigen has been produced and purified.

To control for cross-reactivity, affinity purified antibodies are tested for sensitivity and specificity on protein arrays consisting of glass slides with spotted PrEST fragments.

Antibody specificity is analyzed using Western blot in a standardized setup. Total protein lysates from a limited number of tissues (liver and tonsil), cell lines (RT4 and U-251 MG), and human plasma are used to evaluate the antibody target binding in a Western blot setting. Antibodies with an uncertain standard Western blot are reanalyzed using an over-expression lysate as a positive control.

Immunohistochemical staining of normal and cancer tissue is examined and annotated by specially educated personnel, and the staining patterns are compared with available gene/RNA/protein characterization data.

High resolution confocal microscopy images of human cell lines stained by indirect immunofluorescence are annotated for subcellular localizations by trained cell biologists, and the subcellular localization patterns are compared with the immunohistochemical staining and available experimental protein characterization data.

Clinical data

Policy information about clinical studies
All manuscripts should comply with the ICMJEguidelines for publication of clinical research and a completedCONSORT checklist must be included with all submissions.

Clinical trial registration	N/A
Study protocol	Not a clinical trial. All studies included were observational studies.<br>A discovery proteomics screen was first performed in a nested case/control study (48+48 patients) derived from a cohort of patients with suspected acute VTE, where patients where VTE could be excluded following diagnostic workup served as controls (VEBIOS ER). One target overlapping with a published dataset of proteins associated with prior risk of VTE in a case/control study, with healthy controls derived from the population, (VEBIOS Coagulation), was selected for further study. Target replication was performed in 1) a cohort of patients with suspected acute VTE (DFW-VTE), a case/control study of newly diagnosed patients with VTE, sampled during treatment initiation, using hospital based controls (FARIVE), and a case/control study of patients with prior VTE using population based healthy controls (RETROVE). Testing for assocation of target with recurrent VTE was performed in a case-only sample set of patients with prior VTE followed up to 12 years following the event (MARTHA).
Data collection	VEBIOS ER: Setting and sampling for biobank - Emergency Department of Karolinska University Hospital Solna, Sweden. Recruitment period December 2010 to September 2013 . Data collection from study inclusion questionare together with retrieval from hospital records following discharge. Routine laboratory tests (e.g CRP) was retrieved from hospital data system. Proteomics screen performed in SciLifeLab Plasma Profiling Facility. Biomarker measurements on biobanked samples performed in SciLifeLab research laboratory<br>VEBIOS Coagulation: Setting and sampling for biobank - Coagulation Unit outpatient clinic at the Karolinska University Hospital (Sweden). Recruitment period January 2011 to December 2017. Data collected from questionare at time of inclusion by research nurse. Routine laboratory tests performed at the Hospital Clinical Chemistry laboratory. Biomarker measurements on biobanked samples performed at SciLifeLab research lab<br>DFW-VTE: Setting and sampling for biobank.- Emergency Department at Karolinska University Hospital Huddinge. Recruitment period April 2014 and May 2015. Data collection from hospital records. Routine lab tests performed at Hospital Clinical Chemistry Laboratory on fresh samples. Biomarker measurements on biobanked samples performed at SciLifeLab research lab<br>FARIVE: Setting and sampling for biobank - multicentre case-control study, in patients and outpatients. Recruitment period 2003-2009. Data collection through study questionarie. Biomarker measurements performed in ScilifeLab research lab<br>RETROVE: Setting and sampling for biobank - Hospital de la Santa Creu i Sant Pau of Barcelona (Spain). Recruitment period between 2012 and 2016.<br>MARTHA: Recruitment in Timone Hospital in Marseille (France) and is still ongoing. Patients included in current included between 1994 and 2008. Biomarker measurements at SciLifeLab research lab.
Outcomes	Clinical diagnosis of DVT and/or PE (acute, previous or recurrent VTE)

Flow Cytometry

Plots

Confirm that:

- [x] The axis labels state the marker and fluorochrome used (e.g. CD4-FITC).
- [x] The axis scales are clearly visible. Include numbers along axes only for bottom left plot of group (a 'group' is an analysis of identical markers).
- [x] All plots are contour plots with outliers or pseudocolor plots.
- [x] A numerical value for number of cells or percentage (with statistics) is provided.

Methodology

Sample preparation	Blood was drawn from healthy volunteers free from any anti-platelet therapy for at least 10 days and anticoagulated with sodium citrate or hirudin. All donors signed informed consent, in accordance with approval of the Human Ethics Committee of the Medical University of Vienna (EK237/2004) and the Declaration of Helsinki. Whole blood was centrifuged (120 g, 20 minutes, room temperature) and platelet-rich plasma (PRP) harvested. To obtain isolated platelets, PRP was diluted with PBS and treated with PGI2 (100 ng/ml), centrifuged for 90 sec at 3000 x g and platelets were resuspended in PBS. This step was repeated twice. Platelet-rich plasma (PRP) or isolated platelets were incubated with recombinant CFHR5 (rCFHR5) in PBS (6 µg/ml, 3845-F5, R&D systems) or PBS alone for 10 minutes before treatment with varying concentrations of ADP (1-5 µM), TRAP-6 (3-15 µM) or convluxin (1-6 ng/ml) for 15 minutes. Platelets were subsequently incubated with primary antibodies: anti-human CD62P-AF647 (AK4), anti-human CD63-PE (H5C6) or anti-human CD41/CD61-FITC (PAC-1) (all Biolegend) for 20 minutes, washed (PBS then 500 g for 10 minutes), then fixed with 1 % paraformaldehyde and incubated with Alexa Fluor 647-streptavidin (Jackson Immuno Research, Ely, UK) for 20 minutes. In some experiments PRP was incubated for 20 minutes with 0.25% DMSO, 100µM compstatin, PBS, 10µg/ml anti-C3a/C3a (desArg)(clone K13/16), prior to assay as described above.
Instrument	(Cytoflex, Beckman Coulter GmbH, Krefeld, Germany)

Software	Cytexpert v2.5 (Beckman Coulter GmbH, Krefeld, Germany).
Cell population abundance	The abundance of the platelets were more than 99%, determined by flow cytometry according to their forward and side scatter.
Gating strategy	First, platelets were identified according to their forward (FSC) and side scatter (SSC, x-axis of all blots). More than 99% of the cell population were platelets. Subsequently, expression of different platelet activation markers (CD62P, Pac-1, CD63) were determined.

☒ Tick this box to confirm that a figure exemplifying the gating strategy is provided in the Supplementary Information.
